# *In vitro* and *in-vivo* exploration of physostigmine analogues to understand the mechanistic crosstalk between Klotho and targets for epilepsy

**DOI:** 10.3389/fphar.2025.1580943

**Published:** 2025-04-25

**Authors:** Mansi Dahalia, Haya Majid, Mohd Junaid Khan, Akshat Rathi, Mohd Ashif Khan, Imran Ahmd Khan, Mohammed Samim, Sayeed Ur Rehman, Md Salik Noorani, Divya Vohora, Nidhi

**Affiliations:** ^1^ Department of Translational and Clinical Research, School of Chemical and Life Sciences, Jamia Hamdard, New Delhi, India; ^2^ Department of Pharmacology, School of Pharmaceutical Education and Research, Jamia Hamdard, New Delhi, India; ^3^ Department of Chemistry, School of Chemical and Life Sciences, Jamia Hamdard, New Delhi, India; ^4^ Department of Biochemistry, School of Chemical and Life Sciences, Jamia Hamdard, New Delhi, India; ^5^ Department of Botany, School of Chemical and Life Sciences, Jamia Hamdard, New Delhi, India

**Keywords:** epilepsy, Klotho, physostigmine analogues, neuroinflammation, apoptosis, neuroprotection

## Abstract

**Background:**

Epilepsy and seizures are characterized by neuronal hyperexcitability and damage, influenced by metabolic dysregulation, neuroinflammation, and oxidative stress. Despite available treatments, many patients remain resistant to therapy, necessitating novel therapeutic strategies. Klotho, a neuroprotective, anti-inflammatory, and antioxidative protein has emerged as a potential modulator of epilepsy-related pathways.

**Objective:**

This study investigates the therapeutic potential of novel physostigmine analogues in regulating Klotho expression and its downstream targets in epilepsy.

**Methods:**

An integrative *in vitro* and *in vivo* approach was employed in PTZ-induced kindled mice. Behavioral assessments, including the Morris Water Maze (MWM), Rota Rod, Black and White Box, and Tail Suspension tests were conducted. Biochemical analyses quantified serum glucose, lipid profiles, pro-inflammatory cytokines (TNF-α, FOXO1), and apoptotic proteins (caspase-3). Quantitative real-time PCR (qRT-PCR) was performed to assess Klotho and epilepsy-associated gene expression (STAT3, Bax, Bcl2).

**Results:**

The synthesized physostigmine analogues exhibited varying inhibitory effects on Klotho transcriptional activators, with Compound C (1,8-bis(phenylsulfonyl)-1,8-dihydropyrrolo [2,3-b] indole) showing the weakest inhibition (IC50 = 1.31 µM). *In vivo*, Compound C demonstrated anticonvulsant (p < 0.05), neuroprotective (5 mg/kg, p < 0.05, 10 mg/kg, p < 0.01, 20 mg/kg p < 0.0001), antidepressant (p < 0.05), and anti-inflammatory (p < 0.05) effects in PTZ-induced seizure models, improving motor function (p < 0.001), cognitive performance (p < 0.01), and reducing neuroinflammatory/metabolic markers (p < 0.05), while modulating STAT3 (p < 0.001), BAX (p < 0.001), Bcl2 (p < 0.05), and Klotho (p < 0.05) gene expression.

**Conclusion:**

The therapeutic potential of 1,8-bis(phenylsulfonyl)-1,8-dihydropyrrolo [2,3-b] indole in epilepsy *via* Klotho modulation was observed. Targeting metabolic, inflammatory, and apoptotic pathways presents a promising strategy for epilepsy management. Further research is required to optimize clinical translation and ensure long-term efficacy and safety.

## Highlights

 • Physostigmine Analogues Compound C (PhACC) significantly enhances Klotho expression, reducing neuroinflammation and apoptosis, offering neuroprotection in epilepsy.
 • Compound C exhibits dose-dependent seizure suppression, improves motor coordination, and enhances memory in PTZ-induced kindling and behavioral tests.
 • The compound normalizes glucose, cholesterol, and triglyceride levels while downregulating pro-inflammatory (TNF-α, FOXO1) and apoptotic (caspase-3, BAX/Bcl2) markers.
 • Results suggest PhACC is a promising drug candidate for epilepsy treatment, warranting further pharmacokinetic and clinical investigations.

## 1 Introduction

Epilepsy is a chronic neurological disorder characterized by recurrent, unprovoked seizures resulting from excessive and abnormal neuronal activity in the brain. Affecting nearly 50 million people worldwide, epilepsy remains one of the most prevalent and debilitating neurological conditions (Vetri et al., 2023). Despite advances in antiseizure medications (ASMs) development, approximately 30% of epilepsy patients continue to experience drug-resistant seizures, highlighting the urgent need for novel therapeutic approaches (Kanner and Bicchi, 2022; Khan P. et al., 2024). Emerging evidence suggests that neuroinflammation, oxidative stress, and neuronal excitotoxicity play vital roles in epilepsy pathogenesis, making them promising targets for therapeutic intervention (Klein et al., 2024).

One of the key proteins implicated in neuroprotection and cognitive function is Klotho, a transmembrane protein primarily expressed in the kidney and brain. Klotho has been extensively studied for its role in aging, neurodegeneration, and metabolic disorders, and recent studies suggest that it may also influence seizure susceptibility and neuronal survival (Prud’homme and Wang, 2024). Klotho enhances neuronal survival by inhibiting oxidative stress, reducing neuroinflammation, and promoting synaptic plasticity (Zhao et al., 2024). Studies have shown that Klotho-deficient mice exhibit early aging, cognitive decline, and increased susceptibility to neurodegeneration, while overexpression of Klotho leads to enhanced cognitive function and increased lifespan. In epilepsy, Klotho has been linked to seizure susceptibility and neuronal survival (Gu et al., 2025). Seizures are known to induce oxidative stress and inflammatory responses, contributing to neuronal damage (Wei et al., 2023). Klotho reduces oxidative stress by enhancing the activity of antioxidant enzymes like superoxide dismutase (SOD) and inhibits pro-inflammatory cytokines such as tumor necrosis factor-alpha (TNF-α) and interleukin-6 (IL-6) (Kamali et al., 2021; Khan et al., 2025). Additionally, it has been suggested that Klotho can modulate glutamatergic and cholinergic neurotransmission, thereby regulating neuronal excitability and preventing excessive seizure activity. Due to its neuroprotective, anti-inflammatory, and antioxidant properties, Klotho activation has been proposed as a potential therapeutic strategy for epilepsy and other neurodegenerative conditions. Enhancing Klotho expression may ameliorate cognitive deficits, reduce seizure severity, and improve overall brain health (Gao et al., 2021; Ranjbar et al., 2023).

In the search for potential Klotho activators, Physostigmine, a naturally occurring alkaloid derived from the Calabar bean (*Physostigma venenosum*) has gained attention. It is a reversible inhibitor of acetylcholinesterase (AChE), which is a neurotransmitter crucial for learning, memory, and cognitive function (Tyagi et al., 2023). Due to its ability to enhance cholinergic transmission, physostigmine has been used in the treatment of Alzheimer’s disease, myasthenia gravis, and glaucoma (Kunzler and Erickson, 2022). Unlike other AChE inhibitors, physostigmine readily crosses the blood-brain barrier, making it more effective in treating central nervous system disorders. On the contrary, Physostigmine is also associated with dose-dependent side effects, including nausea, vomiting, bradycardia, and seizures, due to excessive cholinergic stimulation. Another limitation is its poor metabolic stability, leading to rapid degradation and inconsistent therapeutic effects (Arens and Kearney, 2019). Furthermore, while physostigmine effectively inhibits acetylcholinesterase, it lacks additional neuroprotective and anti-inflammatory properties needed for the long-term management of neurodegenerative and epileptic conditions (Rasimas et al., 2018; Nadeem et al., 2025). These challenges highlight the need for structurally modified analogues with enhanced bioavailability, reduced toxicity, and multifunctional therapeutic benefits, such as Klotho activation, anti-inflammatory effects, and better blood-brain barrier permeability. Structurally modified analogues of physostigmine could provide additional pharmacological benefits, particularly in epilepsy management. The development of pyrroloquinoline and pyrroloindoline derivatives may present an innovative approach to designing small molecules that may enhance Klotho transcriptional activity and provide antiepileptic, neuroprotective, and cognitive-enhancing effects (Marta et al., 1988; Imran et al., 2023).

The current study focuses on the synthesis and biological evaluation of pyrroloquinoline and pyrroloindoline-based molecules for their potential effects on Klotho protein activation and neuroprotection in epilepsy models. The present study is based on a multidisciplinary approach, integrating synthetic organic chemistry, *in vitro* transcriptional assays, and *in vivo* behavioral and biochemical evaluations to assess the efficacy of the synthesized compounds on animal models of epilepsy and related neurobehavior.

## 2 Methods

### 2.1 Materials

Pentylenetetrazole (PTZ) was obtained from Sigma-Aldrich, United States, and Sodium Valproate (SVP) was procured as the reference anticonvulsant. All other chemicals used in the study, including dimethyl sulfoxide (DMSO), phosphate-buffered saline (PBS), ethylenediaminetetraacetic acid (EDTA), sodium chloride (NaCl), potassium chloride (KCl), calcium chloride (CaCl_2_), 1% carboxymethyl cellulose (CMC), double-distilled water, and magnesium chloride (MgCl_2_), were of analytical grade and obtained from Amplicon Biotech, New Delhi, India. EDC·HCL, Tempo, Indole-3-carboxyaldehyde, nitroethane, Dulbecco’s Modified Eagle Medium (DMEM), Fetal Bovine Serum (FBS), Triphosgene, nickel (II) bromide, oxalyl chloride, nitromethane, palladium (II) acetate was purchase from SG Enterprises, New Delhi. Enzyme-linked immunosorbent assay (ELISA) kits for biochemical estimations, including inflammatory cytokines TNF-α, Forkhead Box O1 (FOXO1), apoptotic markers cysteine-aspartic protease-3 (Caspase-3), and metabolic markers such as glucose, total cholesterol, triglycerides, and high-density lipoprotein cholesterol (HDL-C), were also procured from Amplicon Biotech. Trizol reagent for ribonucleic acid (RNA) extraction, Cybergreen dye for quantitative real-time polymerase chain reaction (qRT-PCR), and specific primers for gene expression analysis were obtained from Infobiosys Ltd.

### 2.2 *In vitro* study

#### 2.2.1 Synthesis of pyrroloquinoline and pyrroloindoline derivatives for Klotho protein evaluation

The synthesis of compounds A, B, C, and D involved organocatalysis, carbodiimide-mediated coupling, palladium-catalyzed cross-coupling, and oxidative cyclization, demonstrating synthetic versatility. Compound A was synthesized *via* the Henry reaction (nitroaldol reaction), where triethylamine deprotonated nitroethane, generating a nitronate anion that attacked 2-formyl pyrrole, forming a β-nitro alcohol (Kitagaki et al., 2013; Mitchell et al., 2017). The electron-withdrawing nitro group facilitated further transformations, while intramolecular hydrogen bonding influenced its reactivity and potential biological activity. The reaction was conducted using 2-formylpyrrole and nitroethane (1.8 equivalents) in the presence of triethylamine (1.5 equivalents) as a mild base in DMF at 0°C–27°C for 3–5 h. The β-nitro alkoxide intermediate formed during the reaction was protonated to yield the desired β-nitroalcohol with an 85% yield, demonstrating the efficiency of this approach in functionalizing pyrrole derivatives.

Compound B was synthesized via EDC-mediated amide bond formation, where EDC·HCl (1.6 equivalents) activated the carboxyl group of 2-carboxypyrrole, enabling nucleophilic attack by an amine. The reaction was carried out in DMF with triethylamine (2.4 equivalents) at 0°C–27°C for 16 h, leading to the formation of the chloroacetamide derivative. The process achieved an 82% yield, demonstrating the efficiency of EDC-mediated couplings in functionalizing heterocyclic frameworks.

**FIGURE 1:**
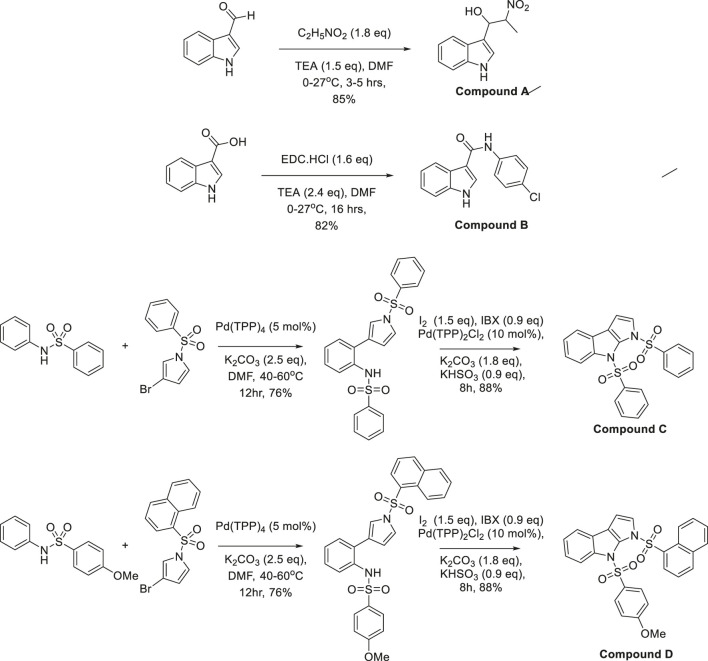
Synthesis of Indolyl-B-EWG (Indolyl beta electron-withdrawing group) system.

Compounds C and D were synthesized via a two-step process involving palladium-catalyzed Buchwald-Hartwig cross-coupling followed by oxidative cyclization (Smith et al., 2008; Zhou et al., 2019). In the first step, Pd (TPP)_4_ (5 mol%) catalyzed the C-N bond formation between a sulfonamide derivative and an aryl bromide in the presence of K_2_CO_3_ (2.5 equivalents) as a base in DMF at 40°C–60°C for 12 h, yielding N-aryl sulfonamide intermediates with 76% efficiency. The second step involved oxidative cyclization using Pd (TPP)_2_Cl_2_ (10 mol%) with IBX (0.9 equivalents) and I_2_ (1.5 equivalents) as oxidants. K_2_CO_3_ (1.8 equivalents) and KHSO_3_ (0.9 equivalents) maintained a basic redox environment, while iodine stabilized transition states. After 8 h of stirring, the reaction produced Compounds C and D in 88% yield. The structural variation between the two resulted from a methoxy substituent in Compound D, which influenced electronic properties, reactivity, high efficiency, excellent yields, and functional group tolerance. (Figure 1, drawn using CHEMDRAW). The synthesized pyrroloquinoline and pyrroloindoline derivatives were characterized using Proton Nuclear Magnetic Resonance (^1^H NMR) spectroscopy to confirm their structural integrity. ^1^H NMR spectra were recorded on a 400 MHz Bruker spectrometer in deuterated dimethyl sulfoxide (DMSO-d_6_) or deuterated chloroform (CDCl_3_) as the solvent, depending on the solubility of the compounds. Chemical shifts (δ) were reported in parts per million (ppm) relative to the internal standard tetramethylsilane (TMS, δ = 0.00 ppm). The spectral data were analyzed for characteristic proton signals, including aromatic, aliphatic, and heterocyclic hydrogen environments. Peak multiplicities were assigned as singlet (s), doublet (d), triplet (t), or multiplet (m), and coupling constants (J values) were measured in Hertz (Hz) to confirm molecular connectivity (de Souza et al., 2025) (Table 1).

**TABLE 1:** IUPAC names of synthesized analogues.

Compounds	Names
Compound A	1-(1H-indol-3-yl)-2-nitropropan-1-ol
Compound B	N-(4-chlorophenyl)-1H-indole-3-carboxamide
Compound C	1,8-bis(phenylsulfonyl)-1,8-dihydropyrrolo [2,3-b]indole
Compound D	8-((4-methoxyphenyl) sulfonyl)-1-(naphthalene-1-ylsulfonyl)-1,8-dihydropyrrolo [2,3-b] indole

#### 2.2.2 Cell culture for Klotho transcriptional activation assay

HEK293T cells were cultured in DMEM supplemented with FBS and a standard mix of penicillin (100 U/mL), streptomycin (100 μg/mL), and amphotericin B (0.25 μg/mL) to prevent contamination without affecting cell viability, at 37°C and 5% CO_2_, with routine *mycoplasma* screening before the experiment. A G418 kill curve was established, identifying 750 μg/ml as the optimal concentration for clone selection. For stable cell line generation, genomic DNA was extracted from HEK293T cells using the PCI method, and the KL promoter (1.8 KB) was amplified via PCR using Hot Star Taq Master Mix Kit (Qiagen) with the following primers:

Forward: 5′-GGG​AAA​TGT​GAT​ACT​CCA​TGT​AGA​CGT​AGC-3′

Reverse: 5′-GCT​GCG​CGG​GAG​CCA​GGC​TCC​GGG​GCC​CCG-3′

The PCR product was cloned into the pCR2.1 vector (Invitrogen) and sub-cloned into pGL3 (Promega) using HindIII and XhoI restriction enzymes. HEK293T cells were co-transfected with the KL promoter and pcDNA3.1 using Lipofectamine™ 2000 (Invitrogen) following the manufacturer’s instructions. Transfected cells were subjected to G418 selection for 10 days. Colonies were isolated by trypsinization and assessed for promoter activity using the luciferase assay system. For the promoter-reporter assay, KL5 stable cells were seeded in 96-well plates, treated with varying concentrations of the compounds for 24 h, and analyzed via luciferase assay. A five-point dose-response curve was generated to evaluate the transcriptional activation of KL by the synthesized molecules.

### 2.3 In vivo: Acute and chronic study

#### 2.3.1 Animals

Healthy Swiss albino mice (25–30 g) of both genders, were sourced from the Central Animal House Facility of Jamia Hamdard, New Delhi, for this study. The mice were housed in polypropylene cages (43 cm × 28.6 cm × 15.5 cm) under regulated conditions, including a 12-h light-dark cycle, temperature of 22°C-24°C, and humidity between 50%-60%. They had free access to water and a standardized pellet diet. Before the experiment, the mice underwent a 1-week acclimatization period to reduce stress. All procedures were carried out following the ethical guidelines of the Committee for Control and Supervision of Experiments on Animals (CCSEA), India, and the Institutional Animal Ethics Committee (IAEC) of Jamia Hamdard (Registration no. JH/993/CCSEA, Protocol no. 1925).

#### 2.3.2 Acute study: increasing current electroshock test

The study involved randomly assigning mice into five groups (n = 6 per group). Group I served as the control, while Group II acted as the positive control for the Increasing Current Electroshock Test (ICES) test, receiving SVP at a dose of 200 mg/kg orally (*p.o.*). Groups III, IV, and V were treated with PhACC at doses of 5, 10, and 20 mg/kg *p. o.,* respectively. All treatments were administered 1 h before the ICES test. *In vitro* analysis showed that PhACC is the most effective analogue out of all thus, it was selected for *in vivo* studies. Using an electro-convulsiometer, each mouse was subjected to an electric shock delivered through ear electrodes. The initial current was set at 2 mA and applied as a single pulse train lasting 0.2 s. The current intensity was incrementally increased by 2 mA every 2 s until tonic hind limb extension (HLE) occurred, which was recorded as the seizure threshold current (STC). If no tonic HLE was observed up to a maximum current of 30 mA, further increases in current were not applied, and this maximum value was used for subsequent calculations (Dahalia et al., n. d.; Jain et al., 2010).

#### 2.3.3 Chronic study

The experimental design involved randomly assigning animals into six distinct groups, each comprising nine mice (Table 2). The sample size was calculated using G* Power software, version 3.1. The groups were as follows:

⁃ Group I (Control): 1% CMC administered orally, and normal saline administered intraperitoneally.
⁃ Group II: 1% CMC administered orally along with PTZ (25 mg/kg, *i. p.*) administered every other day.
⁃ Group III (Standard): SVP at 200 mg/kg *p. o* administered orally in addition to PTZ (25 mg/kg, *i. p.*)
⁃ Group IV: PhACC at 5 mg/kg *p. o* c and PTZ (25 mg/kg, *i. p.*)
⁃ Group V: PhACC at 10 mg/kg *p. o* administered orally along with PTZ (25 mg/kg, *i. p.*)
⁃ Group VI: PhACC at 20 mg/kg *p. o* administered orally along with PTZ (25 mg/kg, *i. p.*)

**TABLE 2:** The number of animals per group as per experiments.

Study type	Number of mice per group	Mice used for ELISA	Mice used for Qrt-PCR
Increasing Current Electroshock Test (ICES)	6	Nil	Nil
Chronic PTZ Study	9	6	3

#### 2.3.4 Drug administration protocol

PhACC was weighed and suspended in 1% CMC using double-distilled water. PTZ was prepared by dissolving it in normal saline (0.9% NaCl solution). Control animals were given 1% CMC suspension in double-distilled water, administered orally once daily. PhACC was provided in doses of 5, 10, and 20 mg/kg, *p. o*., administered orally each day for 35 days. In the PTZ-kindling model, a sub-convulsant dose of PTZ (25 mg/kg*, i. p.*) was administered every other day over the course of 35 days. All drugs were administered at a volume of 10 mL/kg.

#### 2.3.5 Pentylenetetrazole-induced kindling in mice

After administering PTZ, the animals were observed continuously for 30 min, with seizure intensity evaluated at regular intervals using the Racine scale. Seizure severity was classified into the following stages: Stage 0 (no response), Stage 1 (ear and facial twitching), Stage 2 (myoclonic jerks without standing), Stage 3 (upright posture accompanied by bilateral forelimb clonus), Stage 4 (tonic-clonic seizures), and Stage 5 (generalized tonic-clonic seizures with loss of postural control). Mice were deemed fully kindled after exhibiting Stage 4 seizures during three consecutive PTZ administrations. Cumulative seizure scores for each group were calculated on days 7, 14, 21, 28, and 35, facilitating a progressive assessment of seizure progression and the effects of treatment over time (Dahalia et al., 2025).

#### 2.3.6 Behavioral assessment

##### 2.3.6.1 Rotarod test

The rotarod test was used to assess the acute effects of PhACC on skeletal muscle strength, motor coordination, and balance in mice. The apparatus consisted of a rotating rod with a diameter of 3 cm, positioned 25 cm above the base. Each mouse was placed individually on the rod, which rotated at a constant speed of 20 revolutions per minute. The time taken for each mouse to fall off the rod (latency) was measured in seconds, serving as an indicator of muscle strength and motor coordination. Each mouse underwent three consecutive trials, and the average latency was calculated and normalized to body weight (seconds per gram). The apparatus was cleaned between trials to maintain consistent testing conditions (Dunham and Miya, 1957; Herrera et al., 2024).

##### 2.3.6.2 Tail suspension test

The tail-suspension test (TST) was conducted to assess depression-like behavior in mice. This method is based on the observation that mice suspended by their tails exhibit periods of immobility, interpreted as behavioral despair. For the test, mice were suspended 50 cm above the ground by securing approximately one-quarter of their tails with adhesive tape attached to an iron hoop. Each mouse was observed for a total duration of 7 min, with immobility time recorded during the final 6 min. Immobility was defined as the absence of any voluntary limb or body movement, excluding those related to respiration (Steru et al., 1985; Yang et al., 2023).

##### 2.3.6.3 Black and white box test

The black-white box test was used to evaluate anxiety-like behavior in mice, utilizing their natural preference for dark spaces and their spontaneous tendency to explore novel environments (Teixeira et al., 2011). The apparatus consisted of a wooden box measuring 40 cm in length, 15 cm in width, and 15 cm in height, divided equally into two compartments—one dark and one light—connected by a small door that allowed mice to move between them. Each mouse was placed at the doorway facing the dark compartment, and its behavior was observed and recorded during a 10-min trial. The amount of time spent in the light compartment was analyzed as a measure of anxiety-like behavior (Crawley and Goodwin, 1980; Kajita and Mushiake, 2024).

##### 2.3.6.4 Morris water maze test

The Morris Water Maze (MWM) test was conducted to assess memory and learning abilities. The MWM setup consisted of a circular pool divided into four quadrants, measuring 90 cm in diameter and 35 cm in height. A hidden platform was placed in the target quadrant, submerged 1.0–2.0 cm below the water surface, with the water temperature maintained at 22°C–25°C. The test was initiated within 48 h after the final injection and included two phases: spatial memory training and a probe trial.

During the spatial memory training phase, mice were trained four times per day over seven consecutive days. For each trial, mice were placed into the water facing the wall of the pool from four distinct starting points corresponding to the four quadrants and allowed 1 min to locate the hidden platform. Upon reaching the platform, mice remained on it for 10 s. If a mouse failed to find the platform within 1 min, it was guided to the platform and allowed to remain there for 10 s. The time taken to locate the platform (escape latency) was recorded.

The probe trial was conducted 24 h after the last training session. The platform was removed, and mice were released into the quadrant opposite the former platform location, allowing them to swim freely for 60 s. The mean swimming speed in the target zone and the percentage of time spent in each quadrant were recorded as indicators of memory retention and spatial learning. Following the test, all mice were dried with a towel and placed in a warming cage to prevent hypothermia (Morris, 1984; Othman et al., 2022; Zwierzyńska and Pietrzak, 2022).

### 2.4 Biochemical estimations

#### 2.4.1 Tissue preparation and biochemical analysis

Following neurobehavioral assessments, the animals were euthanized for tissue preparation and biochemical analysis. Before sacrifice using a carbon dioxide (CO_2_) chamber with a flow rate that displaced 30%–70% of the chamber’s volume per minute, blood samples were collected from the tail vein (AVMA Guidelines for the Euthanasia of Animals: 2020 Edition | OLAW, n. d.; Gent et al., 2018; Salvati et al., 2022). The serum was separated and stored for the measurement of glucose, insulin, total cholesterol (TC), triglycerides (TG), and high-density lipoprotein cholesterol (HDL-C) levels. Post-euthanasia, the whole brain was extracted and rinsed with cold 0.9% saline. The hippocampus and cerebral cortex were then dissected, weighed, and immediately stored at −80°C for subsequent biochemical analyses. These tissues were homogenized in 0.1 M phosphate-buffered saline (PBS, pH 7.4) to produce a 10% homogenate, keeping the samples on ice throughout the process. The homogenates were centrifuged at 10,000 rpm for 20 min at 4°C. The centrifuge used was the Thermo Fisher Scientific Sorvall Legend Micro 17R and the resulting post-mitochondrial supernatant was utilized to quantify inflammatory cytokines (FOXO1, TNF-α, and Caspase3) using ELISA kits. All assays were performed according to the manufacturer’s protocols (Khan W. U. et al., 2024; Dahalia et al., 2025).

### 2.5 Quantitative real-time polymerase chain reaction

RNA was isolated from snap-frozen tissue stored at −80°C, which was pulverized in liquid nitrogen and homogenized in 1 mL Trizol. After reaching room temperature, centrifugation (10,000 RPM, 10 min, 4°C) removed debris, followed by phase separation using 400 µL chloroform, vortexing (5 min), incubation (RT, 5 min), and centrifugation (10,000 RPM, 10 min, 4°C). The RNA-containing aqueous phase was collected, precipitated with 500 µL cold isopropanol (ice, 15 min), and centrifuged (10,000 RPM, 10 min, 4°C). The pellet was washed with 1,000 µL of 75% ethanol, centrifuged (7,500 RPM, 5 min, 4°C), air-dried, and dissolved in 50 µL nuclease-free water. RNA was heated at 55°C for 10 min and stored at −80°C. Quality was assessed via NanoDrop (A260/A280: 1.8–2.0) and gel electrophoresis.

cDNA synthesis was performed using the Takara PrimeScript™ RT Reagent Kit with 1–5 µg RNA in a 20 µL reaction mixture containing 4 µL 5X buffer, 1 µL dNTP mix, 1 µL Oligo dT primer, 1 µL RTase, 0.5 µL RNase inhibitor, and nuclease-free water. The reaction conditions included annealing (25°C, 5 min), reverse transcription (42°C–50°C, 30–60 min), and enzyme inactivation (85°C, 5 min). cDNA was stored at −20°C or −80°C. Its quality was validated using GAPDH primers via PCR and gel electrophoresis.

PCR reactions (10 µL) contained 5 µL master mix, 1 µL each of forward and reverse primers, 0.5 µL cDNA, and nuclease-free water. Thermal cycling conditions were initial denaturation (95°C, 3 min), 35 cycles of denaturation (95°C, 30 s), annealing (55°C–60°C, 30 s), extension (72°C, 1 min), and a final extension (72°C, 5 min). Primer sequences for Bax, BcL2, Klotho, Stat3, and Beta Actin were designed using NCBI BLAST and validated via gel electrophoresis at 64.1°C.

For qRT-PCR, reactions (10 µL) included 5 µM forward primer, 0.5 µM reverse primer, 1.5 µL cDNA, 5 µL SYBR Green Fast master mix, and nuclease-free water. The protocol involved initial denaturation (95°C, 5 min), 40 cycles of denaturation (94°C, 15 s), annealing (63°C, 1 min), and extension (72°C, 30 s) using a QuantStudio Cycler. Gene expression was analyzed via the 2^−ΔΔCt method, using mouse Beta Actin as the reference gene. Mean values were calculated per group, and relative fold changes were recorded (Ishqi et al., 2016). Primer sequences were sourced from published studies.

STAT3-F: ATG​TTG​GAG​CAG​CAT​CTT​CAG​GAT.

STAT3-R: CTG​CAT​GTC​TCC​TTG​GCT​CTT​GA.

BCL2-F: ATG​TGT​GTG​GAG​AGC​GTC​AAC​AG.

BCL2-R: CAT​ATA​GTT​CCA​CAA​AGG​CAT​CCC.

BAX-F: ATG​AAC​TGG​ACA​GCA​ATA​TGG​AGC.

BAX-R: CAG​TTG​AAG​TTG​CCA​TCA​GCA​AAC​A.

KLOTHO-F: ATG​AAG​TTC​CGC​CAA​TTG​GAG​TCT.

KLOTHO-R: ATC​ATC​CCT​TTT​GGT​GGT​TCC​CG.

### 2.6 Statistical analysis

The experimental results are expressed as the Mean ± Standard Error of the Mean (SEM). Statistical analysis was performed using one-way ANOVA, followed by Tukey’s *post hoc* multiple comparison test. All analyses were conducted using GraphPad Prism nine software, with a p-value <0.05 considered statistically significant.

## 3 Results

### 3.1 Synthesis of Pyrroloquinoline and pyrroloindoline derivatives for Klotho protein evaluation and effect of synthesized physostigmine analogues on Klotho transcriptional activators

All the compounds were synthesized and characterized using proton NMR and mass spectra. The obtained suggested successful synthesis of the desired molecules in good to moderate yield. The NMR spectra of all four compounds are attached in Supplementary File 1.

The inhibitory effects of the synthesized physostigmine analogues on luciferase activity were evaluated in stable HEK cells, and their IC50 values were calculated based on a five-point dose-response curve. The IC50 values for the compounds are presented in Table 1. Among the four compounds tested, Compound C demonstrated the lowest potency, with an IC50 value of 1.31 µM, indicating its weakest inhibitory effect on luciferase activity. This was followed by Compound A, which showed an IC50 value of 0.57 µM, reflecting moderate activity. Compound B and Compound D exhibited relatively lower potencies, with IC50 values of 1.09 µM and 0.23 µM, respectively.

**FIGURE 2:**
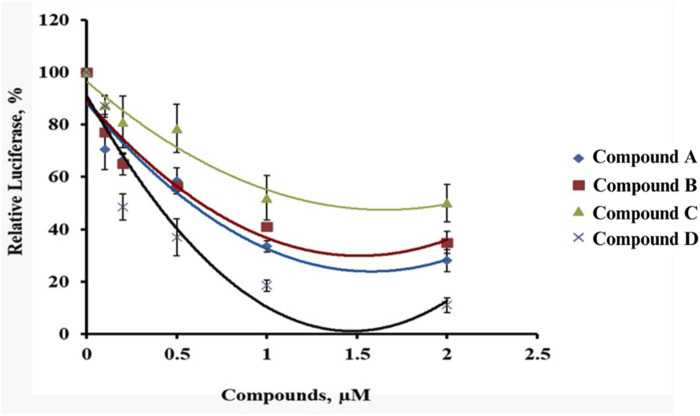
Five-point dose curve showing luciferase activity variation in HEK cells treated with synthesized compounds.

The dose-response curves revealed distinct variations in luciferase activity across the compounds (Figure 2). Comp3 showed a low decline in luciferase activity, achieving inhibition at the highest concentrations, consistent with its low IC50 value. Compound A demonstrated a sharp reduction in luciferase activity, though its curve was slightly less steep than Compound D. In contrast, Compound B and Compound C displayed relatively gradual decreases in activity, reflecting their higher IC50 values and reduced inhibitory potency. It was observed that Compound C was the weakest inhibitor among the synthesized analogues, with the highest IC50 value, followed by Compound B, while Compound A and Compound D showed higher inhibitory effects. These findings suggested the potential for Compound C as the lead compound for further development (Table 3).

**TABLE 3:** IC50 values of synthesized physostigmine analogues.

S. No.	Compound	IC50 (µM)
1	A	0.57
2	B	1.09
3	C	1.31
4	D	0.23

### 3.2 Effect of physostigmine analogue (compound C) administration on increasing current electroshock test

**FIGURE 3:**
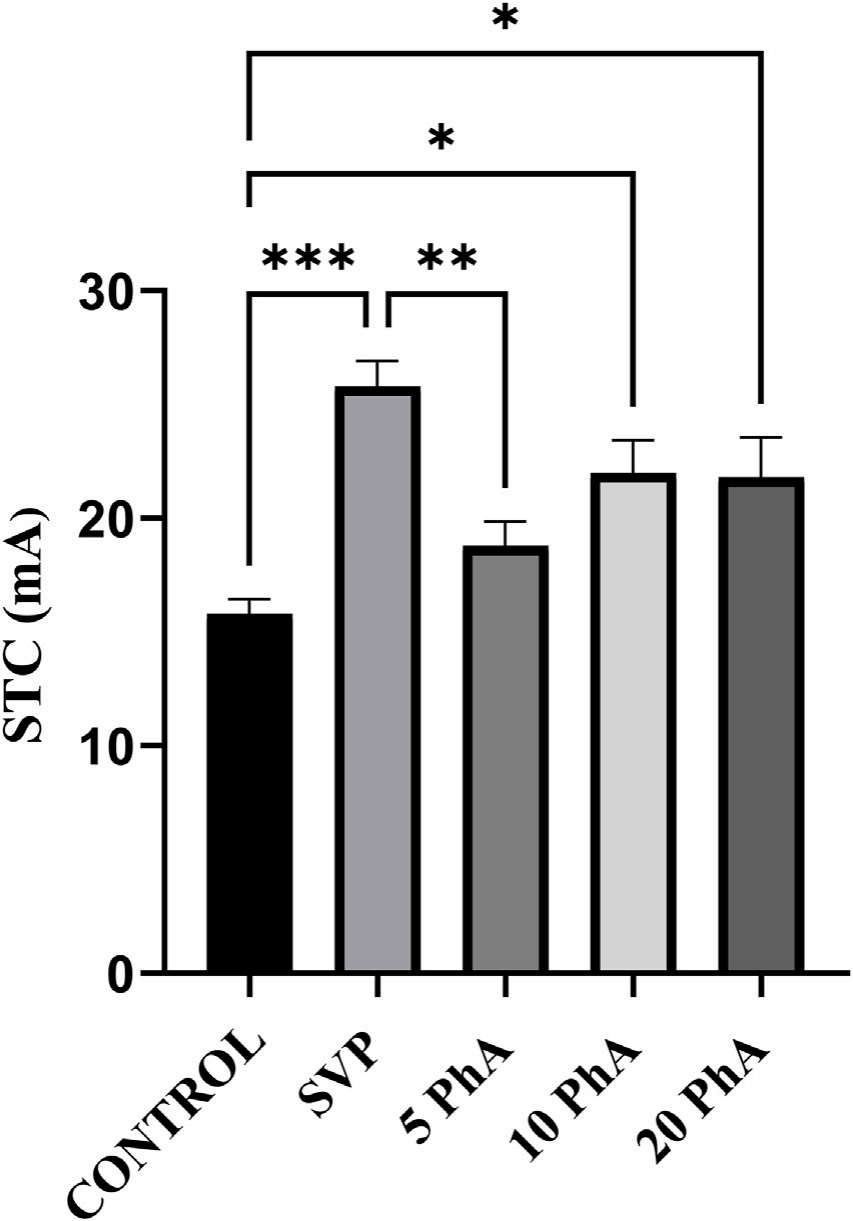
Effect of Physostigmine Analogue Compound C Administration on ICES test. Abbreviations: SVP: Sodium Valproate; PhA: Physostigmine Analogue Compound C, STC: seizure threshold current. Data were analyzed by one-way ANOVA followed by Tukey’s multiple comparison test (n = 6) and expressed as mean ± SEM. Results were considered significant with p-value ≤0.05 ***p < 0.001 Control vs. SVP + PTZ; **p < 0.01 SVP + PTZ vs. 5 mg/kg, p. o., PhA + PTZ; *p < 0.05 Control vs. 10 mg/kg, p. o., PhA + PTZ; 20 mg/kg, p. o. PhA + PTZ.

The SVP group exhibited the highest STC, indicating strong resistance to electrically induced seizures (p < 0.001) compared to the control group. Treatment with Physostigmine Analogues Compound C (PhACC) at doses (10, and 20 mg/kg, *p. o*) significantly increased STC values compared to the control group, demonstrating a dose-dependent anticonvulsant effect. The 10 mg/kg, *p. o.* The PhACC group exhibited a significant increase in STC (p < 0.05) compared to the control group. The 20 mg/kg, *p. o.* The PhACC group also showed a significant increase in STC (p < 0.05) compared to the control group (Figure 3).

### 3.3 Effect of physostigmine analogue compound C administration on pentylenetetrazole-induced kindling model

**FIGURE 4:**
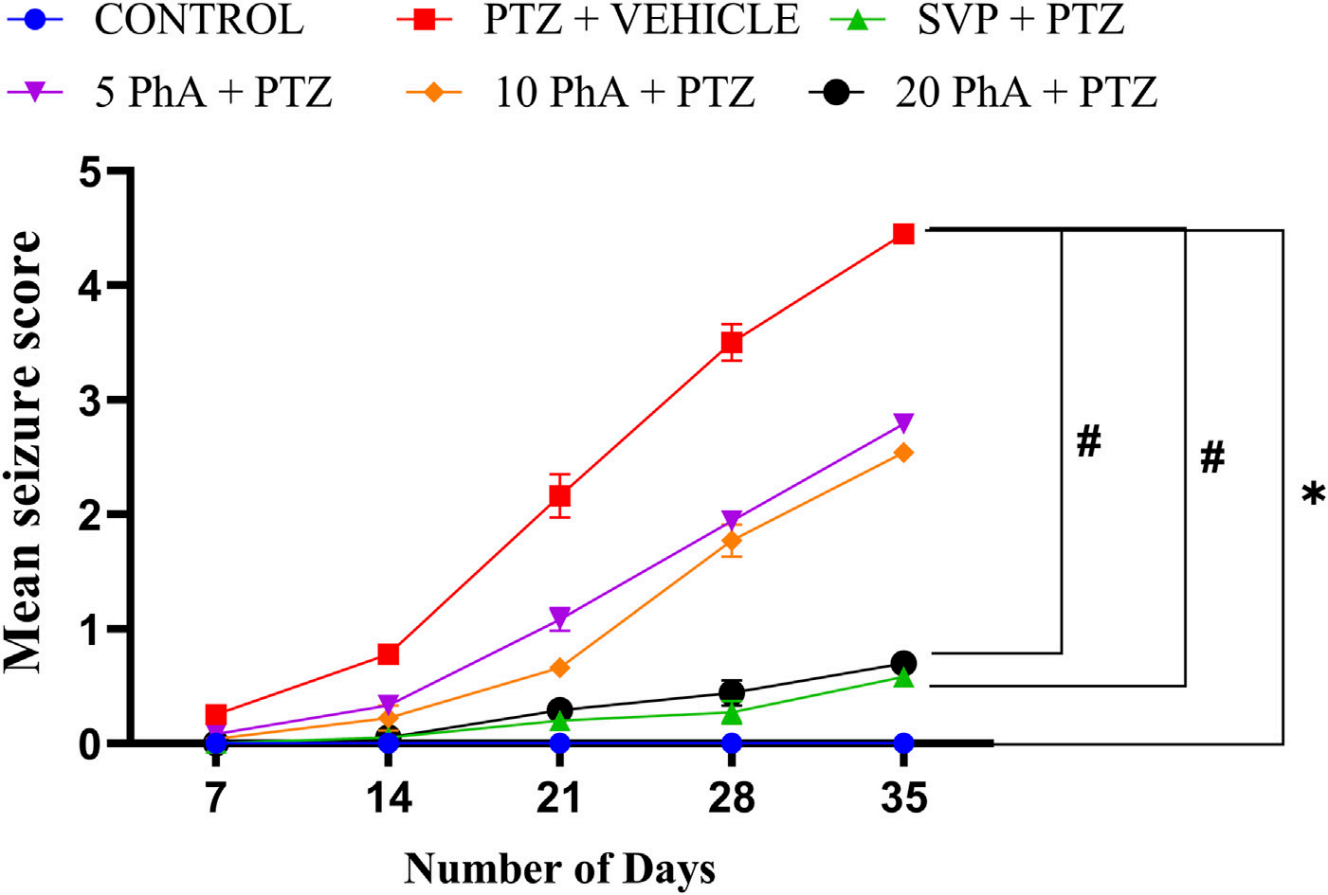
Effect of Physostigmine Analogue Compound C Administration on Pentylenetetrazole-Induced Kindling. Abbreviations: SVP: Sodium Valproate; PhA: Physostigmine Analogue Compound C; PTZ, Pentylenetetrazole. Data were analyzed by one-way ANOVA followed by Tukey’s multiple comparison test and expressed as mean ± SEM (*n* = 9). Results were considered significant with p-value ≤0.05; *p < 0.05 Control vs. PTZ + vehicle, #p < 0.05 PTZ + vehicle vs. SVP + PTZ; 20 mg/kg PhA + PTZ.

Mean seizure scores were calculated by the end of each week over 5 weeks. The group treated with Vehicle + PTZ developed stage 4 seizures by the fifth week, indicating the onset of generalized tonic-clonic seizures. In the first week, all groups started from a relatively similar baseline, with the control group showing stable, low values throughout the study period. In contrast, the groups treated with all three doses of PhACC exhibited a delayed onset of various seizure stages, requiring more days for the progression. Additionally, all experimental mice in the Vehicle + PTZ group were fully kindled. The results demonstrate that PhACC administration in all three doses reduced the mean seizure score in a dose-dependent manner in mice. Control vs. PTZ + Vehicle (p < 0.05), PTZ + Vehicle vs. SVP + PTZ, 20 PhACC + PTZ (p < 0.05) (Figure 4).

### 3.4 Effect of physostigmine analogue compound C administration on rota rod test

**FIGURE 5:**
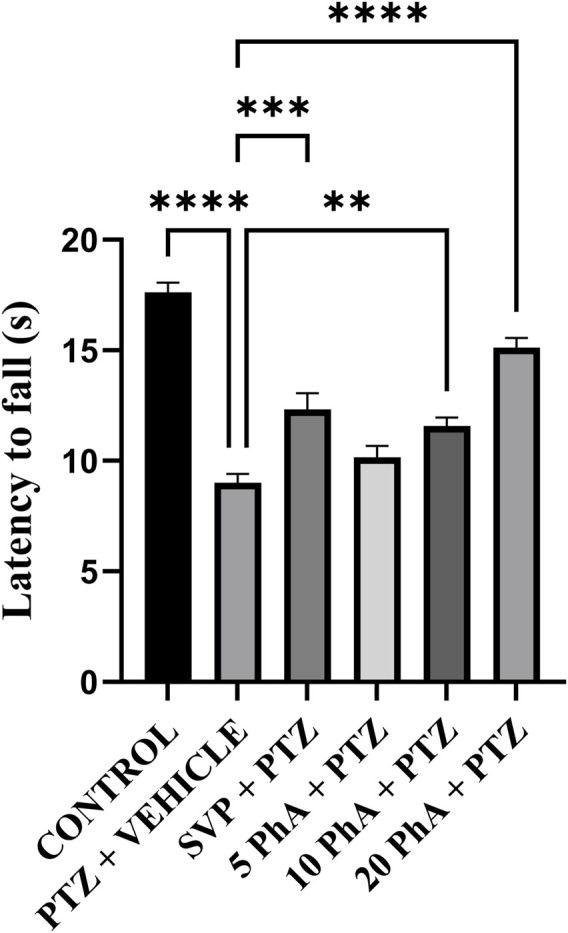
Effect of Physostigmine Analogue Compound C Administration on Rota rod test. Abbreviations: SVP, Sodium Valproate; PhA, Physostigmine Analogue Compound C; PTZ, Pentylenetetrazole. Data were analyzed by one-way ANOVA followed by Tukey’s multiple comparison test (n = 6) and expressed as mean ± SEM. Results were considered significant with p-value ≤0.05 ****p < 0.001 Control vs. PTZ + vehicle, ****p < 0.001 PTZ + vehicle vs. 20 mg/kg PhA + PTZ; ***p < 0.001 PTZ + vehicle vs. SVP + PTZ; **p < 0.01 PTZ + vehicle vs. 10 mg/kg PhA + PTZ.

The PTZ group showed a significant reduction in latency to fall (p < 0.001) compared to the control group. Treatment with SVP (200 mg/kg, *i. p.*) significantly improved latency to fall compared to the PTZ group (p < 0.001). The PhA-treated groups (10 mg/kg *p. o.*, and 20 mg/kg, *p. o.*) also showed a significant improvement in latency to fall compared to the PTZ group. The 10 mg/kg *p. o.,* PhACC group demonstrated a further increase in latency (p < 0.01). The 20 mg/kg, *p. o.,* PhACC group displayed the highest latency to fall (p < 0.001) among the treated groups (Figure 5).

### 3.5 Effect of physostigmine analogue compound C administration on tail suspension test

**FIGURE 6:**
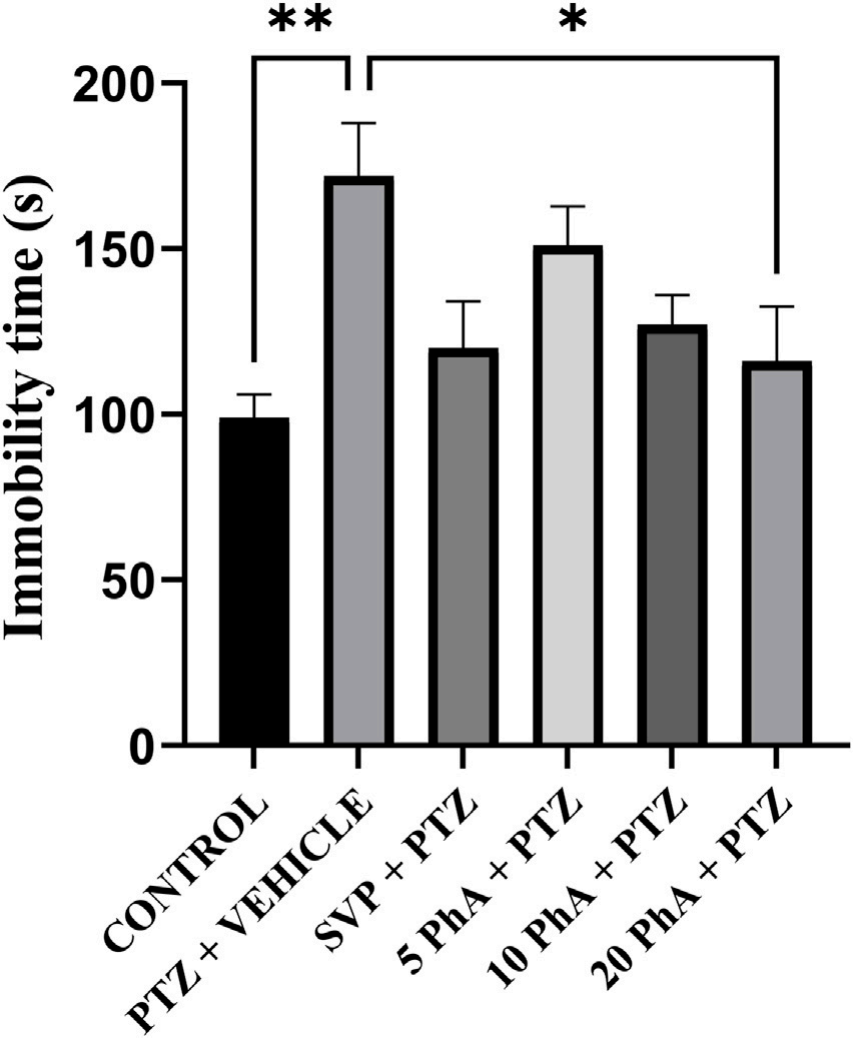
Effect of Physostigmine Analogue Compound C Administration on Tail Suspension Test. Abbreviations: SVP, Sodium Valproate; PhA, Physostigmine Analogue Compound C; PTZ, Pentylenetetrazole. Data were analyzed by one-way ANOVA followed by Tukey’s multiple comparison test (n = 6) and expressed as mean ± SEM. Results were considered significant with p-value ≤0.05 **p < 0.01 Control vs. PTZ + vehicle, *p < 0.05 PTZ + vehicle vs. 20 mg/kg PhA + PTZ.

The PTZ group showed a significant increase in immobility time (p < 0.01) compared to the control group, suggesting depression-like behavior induced by PTZ treatment. Treatment with SVP (200 mg/kg) and PhACC at doses of 5 mg/kg, *p. o.,* and 10 mg/kg, *p. o.* showed an insignificant reduction in immobility time compared to the PTZ group. The PhACC mg/kg, *p. o.* significantly reduced immobility time compared to the PTZ group (p < 0.05). The 20 mg/kg, *p. o.* The PhACC group exhibited the lowest significant immobility time among the PhACC-treated groups (Figure 6).

### 3.6 Effect of physostigmine analogue compound C administration on black and white box test

**FIGURE 7:**
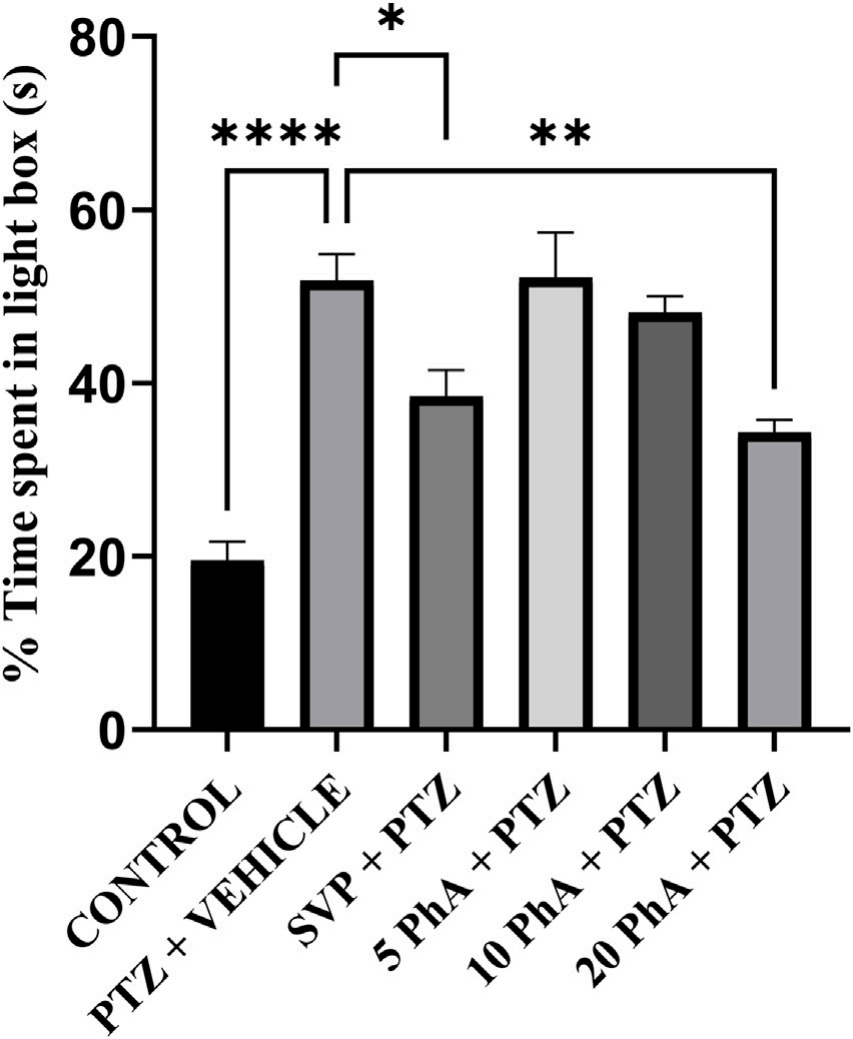
Effect of Physostigmine Analogue Compound C Administration on Black and White box test. Abbreviations: SVP, Sodium Valproate; PhA, Physostigmine Analogue Compound C; PTZ, Pentylenetetrazole. Data were analyzed by one-way ANOVA followed by Tukey’s multiple comparison test (n = 6) and expressed as mean ± SEM. Results were considered significant with p-value ≤0.05 ****p < 0.001 Control vs. PTZ + vehicle; **p < 0.01 PTZ + vehicle vs. 20 mg/kg PhA + PTZ; *p < 0.05 PTZ + vehicle vs. SVP + PTZ.

The PTZ group showed a significant increase in time spent in the light compartment (p < 0.001) compared to the control group, reflecting increased anxiety-like behavior induced by PTZ treatment. The SVP (200 mg/kg, *p. o.*) significantly reduced the time spent in the light compartment compared to the PTZ group (p < 0.05). Similarly, the 20 mg/kg, *p. o.,* PhACC group also demonstrated a significant decrease in time spent in the light compartment among the PhACC-treated groups (p < 0.01) (Figure 7).

### 3.7 Effect of physostigmine analogue compound C administration on morris water maze test

**FIGURE 8:**
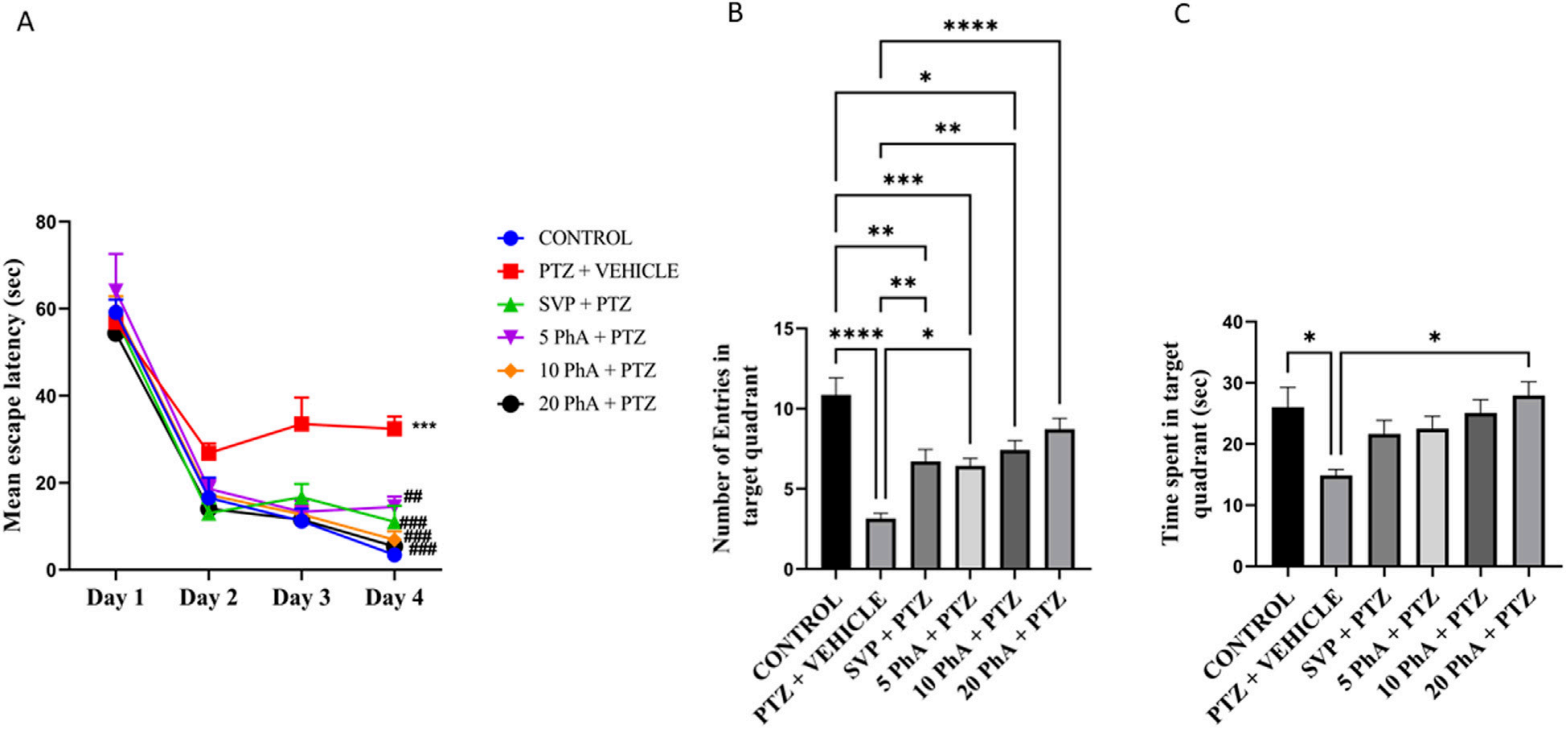
Effect of Physostigmine Analogue Compound C Administration on Morris water maze test. Abbreviations: SVP: Sodium Valproate; PhA: Physostigmine Analogue; PTZ: Pentylenetetrazole. Data were analyzed by one-way ANOVA followed by Tukey’s multiple comparison test (n = 6) and expressed as mean ± SEM. Results were considered significant with p-value ≤0.05 **(A)** Mean escape latency ***p < 0.001 Control vs. PTZ + vehicle, ###p < 0.01 PTZ + vehicle vs. SVP + PTZ. 10 mg/kg PhA + PTZ, 20 mg/kg PhA + PTZ; ##p < 0.05 PTZ + vehicle vs. 5 mg/kg PhA + PTZ. **(B)** Number of entries in the target quadrant, ****p < 0.001 Control vs. PTZ + vehicle, ***p < 0.001 Control vs. 5 mg/kg PhA + PTZ, **p < 0.001 Control vs SVP + PTZ, *p < 0.05 Control vs. 10 mg/kg PhA + PTZ, ****p < 0.001 PTZ + vehicle vs. 20 mg/kg PhA + PTZ, **p < 0.001 PTZ + vehicle vs. 10 mg/kg PhA + PTZ, SVP + PTZ, *p < 0.05 PTZ + vehicle vs. 5 mg/kg PhA + PTZ, **(C)** Time Spent in target quadrant, *p < 0.05 Control vs. PTZ + vehicle, *p < 0.05 PTZ + vehicle vs. 20 mg/kg PhA + PTZ.

The results are presented as (A) Mean Escape Latency, (B) Number of Entries in the Target Quadrant, and (C) Time Spent in the Target Quadrant. A) Mean Escape Latency: On Day 1, all groups demonstrated comparable mean escape latencies (Figure 8). By Day 2–4, the PTZ + Vehicle group exhibited a significantly higher escape latency (p < 0.001) compared to the control group. In contrast, treatment with SVP, PhACC (10, and 20 mg/kg, *p. o.*) (p < 0.001) significantly reduced escape latency over time compared to the PTZ + Vehicle group. The 5 mg/kg, *p. o.* The PhACC group also demonstrated a significant reduction in escape latency compared to the PTZ + vehicle group (p < 0.01). The PTZ + Vehicle group showed a significant reduction in the number of entries into the target quadrant compared to the control group (p < 0.001). Treatment with SVP (p < 0.01) and PhA five (p < 0.05), PhA 10 (p < 0.01), and PhA 20 (p < 0.0001) significantly increased the number of entries into the target quadrant compared to the PTZ group. Among these, the 20 mg/kg PhA group showed the highest number of entries. The PTZ + Vehicle group spent significantly less time in the target quadrant compared to the control group (p < 0.05). The 20 mg/kg, *p. o.* PhACC group exhibited a significantly increased time spent in the target quadrant (p < 0.05).

### 3.8 Effect of physostigmine analogue compound C administration on neuroinflammatory markers in mice

**FIGURE 9:**
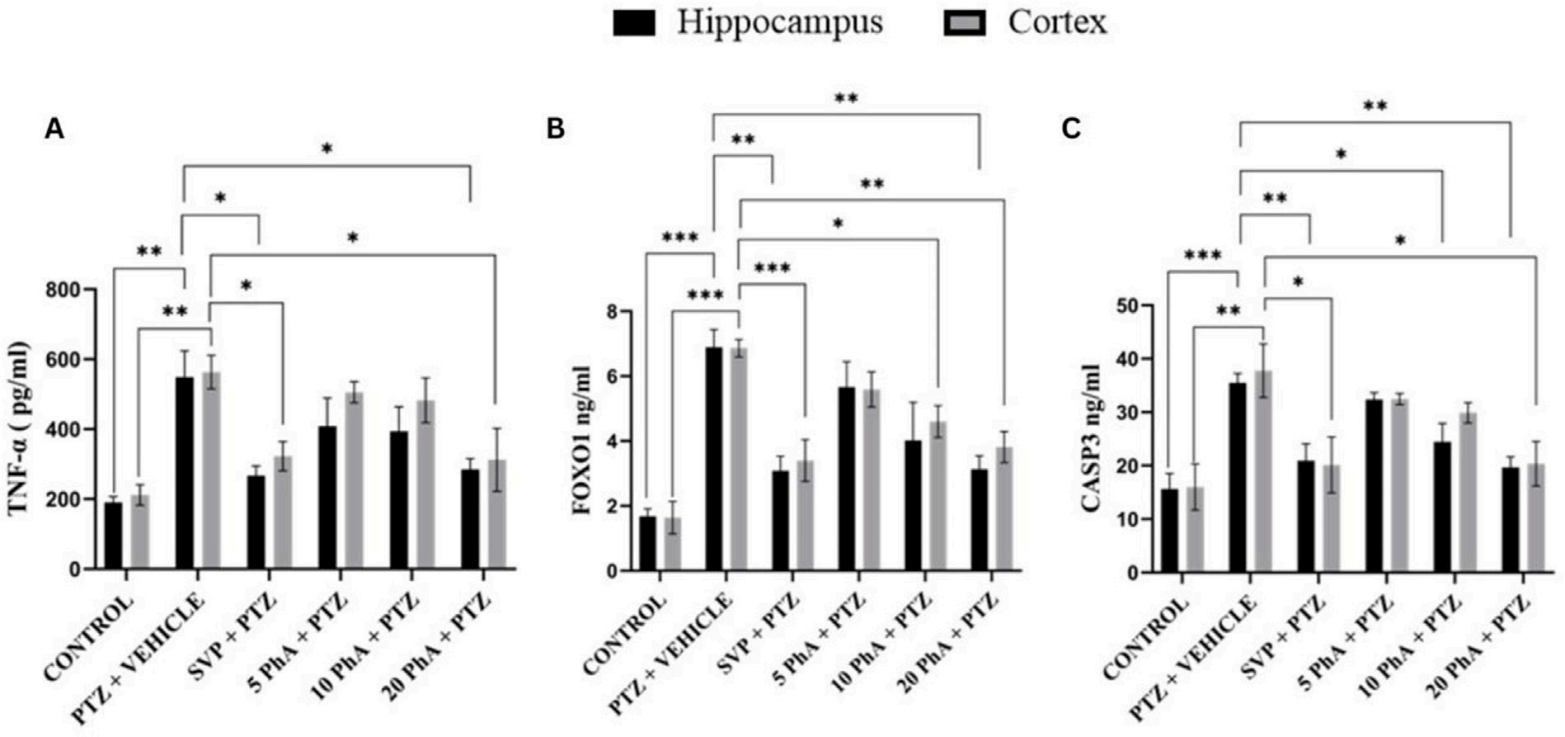
Effect of Physostigmine Analogue Compound C Administration on Neuroinflammatory markers in mice. Abbreviations: SVP, Sodium Valproate; PhA, Physostigmine Analogue Compound C; PTZ, Pentylenetetrazole; TNF-α, umor necrosis factor-alpha, FOXO1, Forkhead Box O1, Caspase-3: apoptotic markers cysteine-aspartic protease-3. Data were analyzed by one-way ANOVA followed by Tukey’s multiple comparison test (n = 6) and expressed as mean ± SEM. Results were considered significant with p-value ≤0.05 **(A)** TNF-α: **p < 0.01 Control vs. PTZ + vehicle, *p < 0.05 PTZ + vehicle vs. SVP + PTZ, 20 mg/kg PhA + PTZ, 20 mg/kg PhA + PTZ. **(B)** FOXO 1: ***p < 0.001 Control vs. PTZ + vehicle, ***p < 0.001 Control vs SVP + PTZ, **p < 0.01 PTZ + vehicle vs. SVP + PTZ, 20 mg/kg PhA + PTZ, *p < 0.05 PTZ + vehicle vs. 10 mg/kg PhA + PTZ, **(C)** Caspase 3:***p < 0.001 Control vs. PTZ + vehicle, **p < 0.01 Control vs. PTZ + vehicle **p < 0.01 PTZ + vehicle vs. SVP + PTZ, 20 mg/kg PhA + PTZ, *p < 0.05 PTZ + vehicle vs. 10 mg/kg PhA + PTZ, 20 mg/kg PhA + PTZ.

The PTZ + Vehicle group exhibited a significant increase in TNF-α levels in both the hippocampus and cortex compared to the control group (p < 0.01). Treatment with SVP 200 mg/kg, *p. o.* significantly reduced TNF-α levels compared to the PTZ + Vehicle group (p < 0.05). Similarly, PhACC treatment at 20 mg/kg, *p. o.* reduced TNF-α levels significantly (p < 0.05) in both brain regions. The PTZ + Vehicle group showed a marked increase in FOXO1 levels in both the hippocampus and cortex compared to the control (p < 0.001). Treatment with SVP (200 mg/kg, *i. p.*) significantly reduced FOXO1 levels (p < 0.01) in the hippocampus and (p < 0.001) in the cortex compared to PTZ + Vehicle. Similarly, PhA 10 mg/kg significantly reduced FOXO1 levels (p < 0.05) in the cortex, whereas the 20 mg/kg, *p. o.* PhACC group exhibited the most significant reduction (p < 0.01) in FOXO1 levels in both the hippocampus and cortex. A significant elevation in Caspase-3 levels was observed in the PTZ + Vehicle group in both the hippocampus (p < 0.001) and cortex (p < 0.01) compared to the control. Treatment with SVP (200 mg/kg, *p. o.*) led to a significant reduction in Caspase-3 levels (p < 0.05) in the cortex and (p < 0.01) in the hippocampus compared to the PTZ + vehicle group. The PhA 10 mg/kg, *p. o.,* group showed a reduction in Caspase-3 levels in only hippocampus (p < 0.05) compared to PTZ + vehicle, whereas PhACC 20 mg/kg, *p. o.,* showed a significant reduction in both cortex (p < 0.05) and hippocampus (p < 0.01) in Caspase-3 levels (Figure 9).

### 3.9 Effect of physostigmine analogue compound C administration on metabolic markers in mice

**FIGURE 10:**
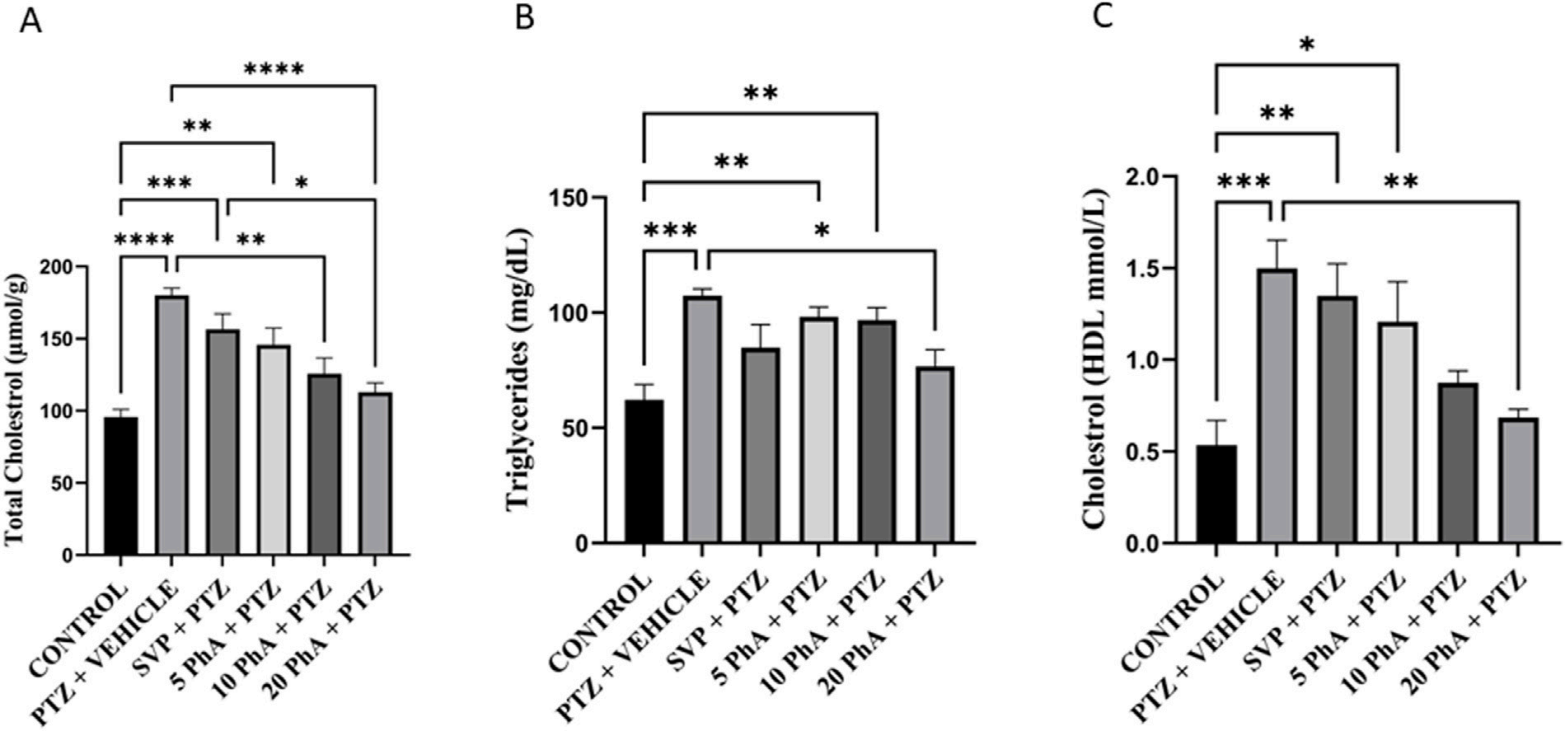
Effect of Physostigmine Analogue Compound C Administration on Metabolic markers in mice. Abbreviations: SVP, Sodium Valproate; PhA, Physostigmine Analogue Compound C; PTZ, Pentylenetetrazole; HDL: High-Density Lipoprotein. ^D^ata were analyzed by one-way ANOVA followed by Tukey’s multiple comparison test (n = 6) and expressed as mean ± SEM. Results were considered significant with p-value ≤0.05 **(A)** Total cholesterol: ****p < 0.001 Control vs. PTZ + vehicle, ***p < 0.001 control vs. 20 mg/kg SVP + PTZ, **p < 0.01 control vs 5 mg/kg PhA + PTZ, ****p < 0.001 PTZ + vehicle vs. 20 mg/kg PhA + PTZ, **p < 0.01 PTZ + vehicle vs. 10 mg/kg PhA + PTZ, **(B)** Triglycerides: ***P < 0.001 Control vs. PTZ + vehicle, **p < 0.01 Control vs. 5 mg/kg PhA + PTZ, 10 mg/kg PhA + PTZ *p < 0.01 PTZ + vehicle vs. 20 mg/kg PhA + PTZ, **(C)** Cholesterol:***p < 0.001 Control vs. PTZ + vehicle, **P < 0.01 Control vs. SVP + PTZ, *p < 0.05 Control vs. 5 mg/kg PhA + PTZ, **p < 0.01 PTZ + vehicle vs. 20 mg/kg PhA + PTZ.

The total cholesterol levels were significantly elevated in the PTZ + Vehicle group compared to the control group (p < 0.001). PhACC treatment at 10 mg/kg, *p. o.*, and 20 mg/kg, *p. o.* doses also showed a significant reduction in cholesterol levels compared to the PTZ group (p < 0.001, p < 0.001), with the 20 mg/kg, *p. o.* dose exhibiting the most substantial improvement. TG levels were markedly increased in the PTZ + Vehicle group compared to the control group (p < 0.001). PhACC treatment at 20 mg/kg, *p. o.* significantly lowered TG levels (p < 0.05). The HDL-C levels were significantly elevated in the PTZ + Vehicle group compared to the control group (p < 0.001). PhACC treatment at 20 mg/kg, *p. o.* demonstrated a dose-dependent reduction in HDL-C levels, with the 20 mg/kg, *p. o.* dose showing significant levels compared to the PTZ group (p < 0.01) (Figure 10).

### 3.10 Effect of physostigmine analogue compound C administration on the glucose levels in mice

**FIGURE 11:**
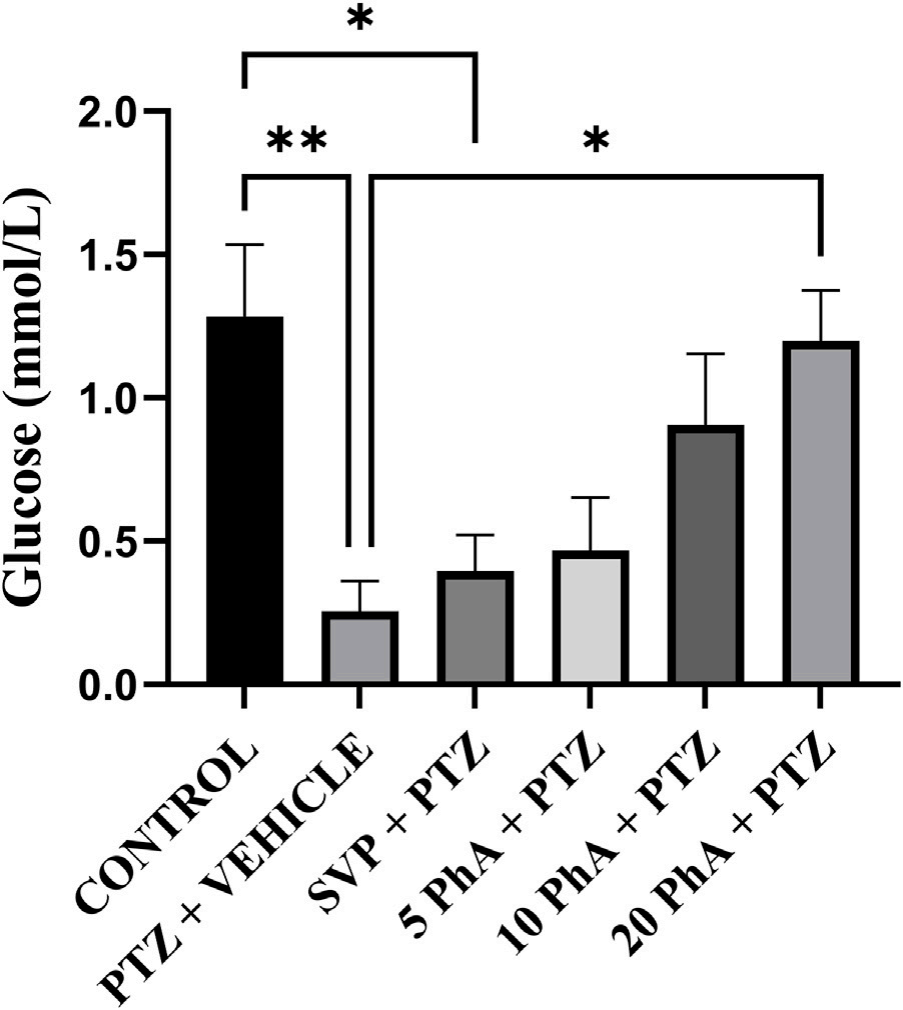
Effect of Physostigmine Analogue Compound C Administration on the Glucose levels in mice. Abbreviations: SVP, Sodium Valproate; PhA, Physostigmine Analogue Compound C; PTZ, Pentylenetetrazole. Data were analyzed by one-way ANOVA followed by Tukey’s multiple comparison test (n = 6) and expressed as mean ± SEM. Results were considered significant with p-value ≤0.05, **p < 0.01 Control vs. PTZ + vehicle, *p < 0.05 control vs. SVP + PTZ, *p < 0.01 PTZ + vehicle vs. 20 mg/kg PhA + PTZ.

The PTZ + Vehicle group exhibited a significant decrease (p < 0.01) in blood glucose levels compared to the control group. Treatment with SVP 200 mg/kg, *p. o*. significantly reduced glucose levels compared to the PTZ group. The SVP + PTZ group also showed a significant decrease in blood glucose levels (p < 0.05) compared to the control group. The PhA 20 mg/kg, *p. o.* showed a significant increase in glucose levels compared to the PTZ group (P < 0.05) (Figure 11).

### 3.11 Effect of physostigmine analogue compound C administration on signal transducer and activator of transcription 3, Bcl2 associated X, apoptosis regulator, B-cell Lymphoma 2, and Klotho gene expression in mice

**FIGURE 12:**
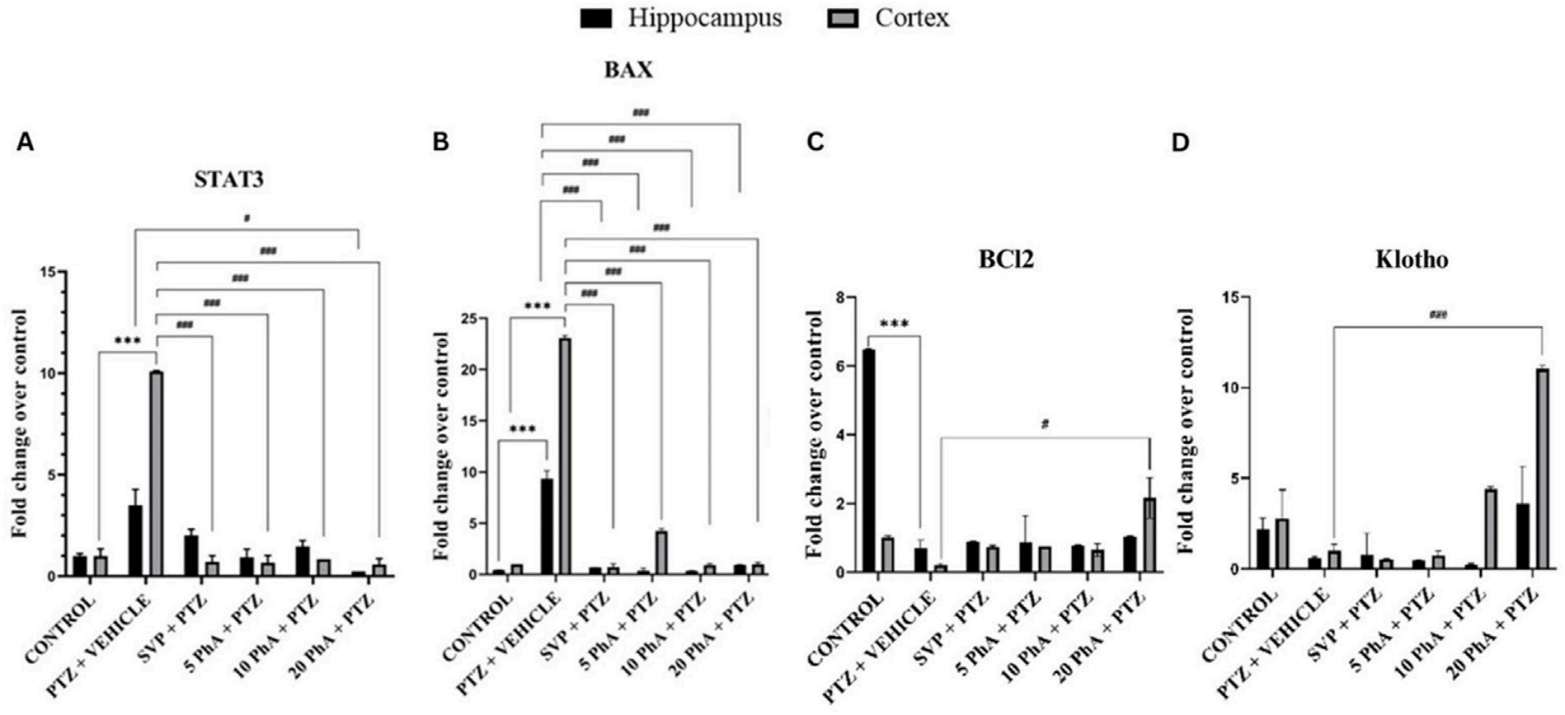
Effect of physostigmine analogue compound C administration on **(A)**: Signal Transducer and Activator of Transcription 3 **(B)**: Bcl2 Associated X Apoptosis Regulator **(C)**: B-Cell Lymphoma 2 **(D)**: Klotho gene expression in mice. Abbreviations: SVP: Sodium Valproate; PhA: Physostigmine Analogue; PTZ: Pentylenetetrazole; Signal Transducer and Activator of Transcription 3 (STAT3); Bcl2 Associated X, Apoptosis Regulator (BAX); B-Cell Lymphoma 2 (Bcl2). Data were analyzed by one-way ANOVA followed by Tukey’s multiple comparison test (n= 3) and expressed as mean ± SEM. Results were considered significant with p-value ≤ 0.05, A) STAT3, ***p < 0.001 Control vs. PTZ + vehicle, ###p < 0.001 control vs. SVP + PTZ, 5 mg/kg PhA + PTZ, 10 mg/kg PhA + PTZ, 20 mg/kg PhA + PTZ, #p < 0.01 PTZ + vehicle vs. 20 mg/kg PhA + PTZ. , B) BAX, ***p < 0.001 Control vs. PTZ + vehicle, ###p < 0.001 control vs. SVP + PTZ, 5 mg/kg PhA + PTZ, 10 mg/kg PhA + PTZ, 20 mg/kg PhA + PTZ, C) BCL2, ***p < 0.001 Control vs. PTZ + vehicle, #p < 0.01 PTZ + vehicle vs. 20 mg/kg PhA + PTZ, D) Klotho, ###p < 0.001 control vs. 20 mg/kg PhA + PTZ.

The fold change expressions of Signal Transducer and Activator of Transcription 3 (STAT3), Bcl2 Associated X, Apoptosis Regulator (BAX), B-Cell Lymphoma 2 (Bcl2), Klotho genes in experimental groups are shown in Figures 12A–D. PTZ + vehicle group showed significantly increased (p < 0.001) STAT3 gene expression in the cortex compared to the control group. 5 mg/kg, *p. o.* PhA + PTZ, 10 mg/kg, *p. o.* PhA + PTZ, 20 mg/kg, *p. o.* PhA + PTZ and SVP + PTZ groups showed a significant decrease (p < 0.001) in STAT3 gene expression in the cortex and in the hippocampus only 20 mg/kg, *p. o.* PhA + PTZ group showed a significant decrease (p < 0.001) in STAT3 gene expression. BAX gene expression was also significantly raised (p < 0.001) in PTZ + vehicle group compared to the control group in both cortex and hippocampus, similarly the BAX gene expression was significantly reduced (p < 0.001) in all the treatment groups 5 mg/kg PhA + PTZ, 10 mg/kg, *p. o.* PhA + PTZ, 20 mg/kg, *p. o.* PhA + PTZ and SVP + PTZ in both the cortex and the hippocampus. The Bcl2 gene expression was significantly decreased (p < 0.001) in the PTZ + vehicle group compared to the control group in the hippocampus and the expression was significantly reduced (p < 0.05) only in 20 mg/kg, *p. o.* PhA + PTZ compared to PTZ + vehicle group in cortex only. The Klotho gene expression was significantly increased (p < 0.05) in 20 mg/kg, *p. o.* PhA + PTZ group compared to PTZ + vehicle group in the cortex.

## 4 Discussion

The present study investigated the potential neuroprotective and anticonvulsant effects of synthesized pyrroloquinoline and pyrroloindoline-based PhACC in ICES and PTZ-induced kindling epilepsy models, with a specific focus on their ability to enhance Klotho transcriptional activity. Our findings provide compelling evidence that PhACC exhibited dose-dependent neuroprotective, anti-seizure, anti-inflammatory, and cognitive-enhancing effects, making it a promising candidate for further development in epilepsy therapy (Marta et al., 1988).

The study demonstrated that among the synthesized analogues, Compound C exhibited the most significant increase in Klotho transcriptional activity in HEK cells, suggesting its potential role in modulating neuroprotection. This is consistent with previous studies showing that Klotho expression is inversely associated with oxidative stress, inflammation, and neuronal damage (Prud’homme et al., 2022; Orellana et al., 2023; Prud’homme and Wang, 2024). Klotho has been shown to protect against oxidative stress by enhancing antioxidant pathways (Orellana et al., 2023). It upregulates nuclear factor erythroid 2-related factor 2 (Nrf2), a key regulator of cellular defense against oxidative stress while inhibiting nuclear factor kappa B (NF-κB), which is involved in inflammation and oxidative damage (Guo et al., 2018). Simultaneously, Klotho gene delivery has been found to suppress NADPH oxidase 2 (Nox2) expression, reducing superoxide production and oxidative damage in vascular tissues (Wang et al., 2012). Additionally, the increase in Klotho gene expression in the cortex of PhACC-treated groups further supports its neuroprotective and cognitive-enhancing properties, particularly in the higher-dose treatment groups. Previously, it was observed that in an amyotrophic lateral sclerosis (ALS) model, Klotho overexpression delayed disease onset, reduced neuronal loss, and decreased inflammation (Zeldich et al., 2019).

The ICES model was used to evaluate seizure susceptibility and resistance in mice. PhACC at 10 mg/kg and 20 mg/kg significantly increased STC, indicating a dose-dependent anticonvulsant effect. The highest dose (20 mg/kg) exhibited a comparable effect to SVP, a widely used ASM. This suggests that PhACC may modulate neuronal excitability, possibly through Klotho-mediated pathways, cholinergic modulation, or oxidative stress reduction (Marta et al., 1988; Wang et al., 2012; Guo et al., 2018; Zeldich et al., 2019). Similarly, in the PTZ-induced kindling model, which mimics chronic epilepsy and seizure progression, PhACC significantly delayed the onset and progression of seizure severity in a dose-dependent manner. Particularly, the 20 mg/kg PhACC treatment group exhibited the most pronounced seizure suppression, suggesting a potential disease-modifying effect rather than just symptomatic control. In the rotarod test, PhACC treatment significantly improved latency to fall, indicating that it does not induce significant motor impairments, unlike some conventional ASMs. The TST and black-and-white box test revealed that PTZ-kindled mice exhibited depression-like and anxiety-like behaviors, which were mitigated by PhACC treatment, especially at the 20 mg/kg dose. This suggested that Klotho activation may have antidepressant and anxiolytic effects as it influences Glutamate [N-methyl-D-aspartate] receptor subunit 2B, which are crucial for synaptic function and plasticity in depression that are consistent with previous findings (Wu et al., 2022).

PhACC treatment significantly improved spatial learning and memory retention, with the 20 mg/kg dose showing the highest efficacy. This improvement aligns with prior evidence that Klotho overexpression enhances cognition in both mice and humans (Abraham et al., 2016). As observed in a Temporal Lobe Epilepsy rat model, Klotho overexpression significantly reduced iron overload, increased glutathione peroxidase-4 (GPX-4) and glutathione (GSH) levels, and decreased reactive oxygen species (ROS) in the hippocampus. This led to improved cognitive function and neuroprotection in epilepsy models (Xiang et al., 2021). Studies suggest that Klotho enhances synaptic function and supports oligodendrocyte maturation, which is crucial for maintaining cognitive abilities (Salech et al., 2019).

Treatment with PhACC 20 mg/kg, significantly reduced TNF-α, FOXO1, and Caspase-3 levels in both the hippocampus and cortex, indicating its anti-inflammatory and neuroprotective properties. This effect is likely mediated through Klotho activation, as it downregulates pro-inflammatory cytokines like TNF-α interleukin-6 (IL-6), and IL-1β while enhancing anti-inflammatory responses (Sedighi et al., 2019; Wei et al., 2021). Klotho activates FOXO1/FOXO3, which helps in reducing oxidative stress, and inflammation and prevents cell apoptosis (Wei et al., 2021), while TNF-α also promotes FOXO1, leading to pro-apoptotic gene expression, including Bim, FasL, and caspases, which drive cell death. Blocking FOXO1 reduces TNF-α-induced apoptosis (Alikhani et al., 2005). FOXO1 activates caspase-3, a key executioner of apoptosis. In TNF-α-induced apoptosis models, FOXO1 silencing reduced caspase-3 activation, preventing excessive cell death (Zhang et al., 2015). In vascular endothelial cells, Klotho downregulates caspase-3 expression, protecting against oxidative stress-induced apoptosis (Cui et al., 2018).

Epilepsy and chronic seizure activity are often associated with metabolic dysregulation, including alterations in lipid metabolism and glucose homeostasis (Devinsky et al., 2018). In this study, PTZ-kindled mice exhibited increased total cholesterol and triglyceride levels, along with reduced glucose levels, reflecting metabolic disturbances. PhACC treatment at 10 mg/kg and 20 mg/kg significantly improved lipid profiles and normalized glucose levels, indicating a potential metabolic stabilizing effect. The involvement of Klotho in insulin signaling and metabolic regulation may explain these findings as Klotho inhibits insulin and IGF-1 signaling by reducing phosphorylation of insulin receptor substrates (IRS-1/2), leading to decreased insulin sensitivity (Raj and Ahuja, 2024). It acts through the PI3K-Akt and MAPK pathways, which are crucial for insulin-mediated glucose uptake (Hasannejad et al., 2019).

Gene expression analysis of STAT3, BAX, Bcl2, and Klotho further supported the neuroprotective role of PhACC. STAT3, a transcription factor involved in neuroinflammation and apoptosis, was significantly upregulated in PTZ-treated mice, consistent with previous reports on its role in epilepsy-induced neuroinflammation. PhACC treatment significantly reduced STAT3 expression, particularly at 20 mg/kg, suggesting its potential inhibition by increasing Bcl-2 expression and reducing BAX (Sun et al., 2017). In this study, the observed increase in BAX, a pro-apoptotic gene, alongside a decrease in Bcl2, an anti-apoptotic gene, in PTZ-kindled mice indicated heightened neuronal apoptosis. However, PhACC treatment effectively restored the BAX/Bcl2 balance, favoring neuronal survival and neuroprotection. Additionally, Klotho gene expression was significantly upregulated in PhACC-treated groups, which may have contributed to STAT3 inhibition and counteracted physostigmine-induced oxidative stress, aligning with previous findings on Klotho’s neuroprotective properties (Cong et al., 2018).

While this study provides strong preclinical evidence for the neuroprotective and anticonvulsant effects of PhACC, several limitations should be addressed. The exact molecular mechanism of Klotho activation by PhACC remains to be elucidated. Further mechanistic studies using Klotho knockdown or overexpression models are needed. The long-term safety and pharmacokinetics of PhACC need to be investigated to assess its potential for clinical translation. Additional epilepsy models, including genetic and traumatic brain injury-induced epilepsy, should be explored to confirm the broad-spectrum efficacy of PhACC. Future studies should evaluate PhACC in combination with existing ASMs to assess possible synergistic effects. Also, studies should optimize the structure-activity relationship (SAR) of physostigmine analogues to enhance their specificity and efficacy for Klotho.

## 5 Conclusion

Through a combination of *in vitro* and *in vivo* approaches, the study observed significant neuroprotective, anti-inflammatory, antioxidative, and anti-apoptotic properties of PhACC. PhACC treatment demonstrated a dose-dependent efficacy in restoring metabolic balance, reducing oxidative stress, and alleviating neuroinflammation and apoptosis. The highest dose (20 mg/kg) consistently showed the most pronounced therapeutic benefits, enhancing motor coordination, reducing anxiety-like and depression-like behaviors, and improving memory and spatial learning. Furthermore, PhACC modulated key biomarkers, including TNF-α, FOXO1, caspase-3, cholesterol, triglycerides, and glucose levels, providing a mechanistic basis for its neuroprotective effects. The study also emphasizes the role of Klotho as a promising therapeutic target in epilepsy due to its multifaceted regulatory functions in metabolic, inflammatory, and oxidative stress pathways. By elucidating the mechanistic crosstalk between Klotho and these pathways, this research opens new avenues for developing innovative strategies to address the challenges of epilepsy and its comorbidities. These findings not only advance our understanding of the molecular mechanisms underlying epilepsy but also pave the way for innovative therapeutic strategies to improve patient outcomes and quality of life.

## Data Availability

The original contributions presented in the study are included in the article/supplementary material, further inquiries can be directed to the corresponding author.
