# Supplementary material for: In vitro and in-vivo exploration of physostigmine analogues to understand the mechanistic crosstalk between Klotho and targets for epilepsy
Source: Front Pharmacol. 2025 Apr 25;16:1580943. doi: 10.3389/fphar.2025.1580943 (PMC12062037; doi:10.3389/fphar.2025.1580943)
Supplement: Supplementary file 1 [file Presentation1.pptx]

## Slide 1
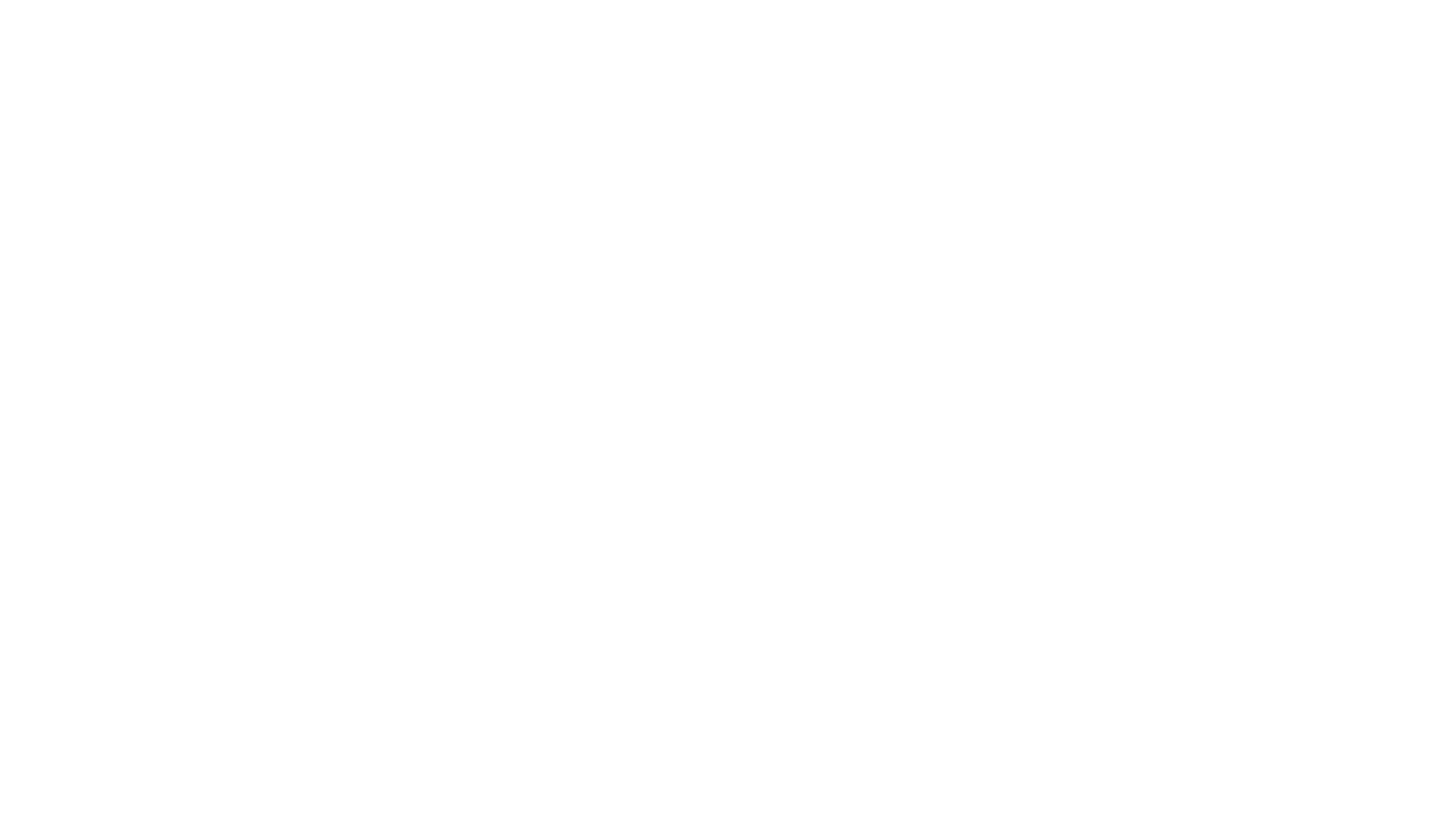

#

## Slide 2
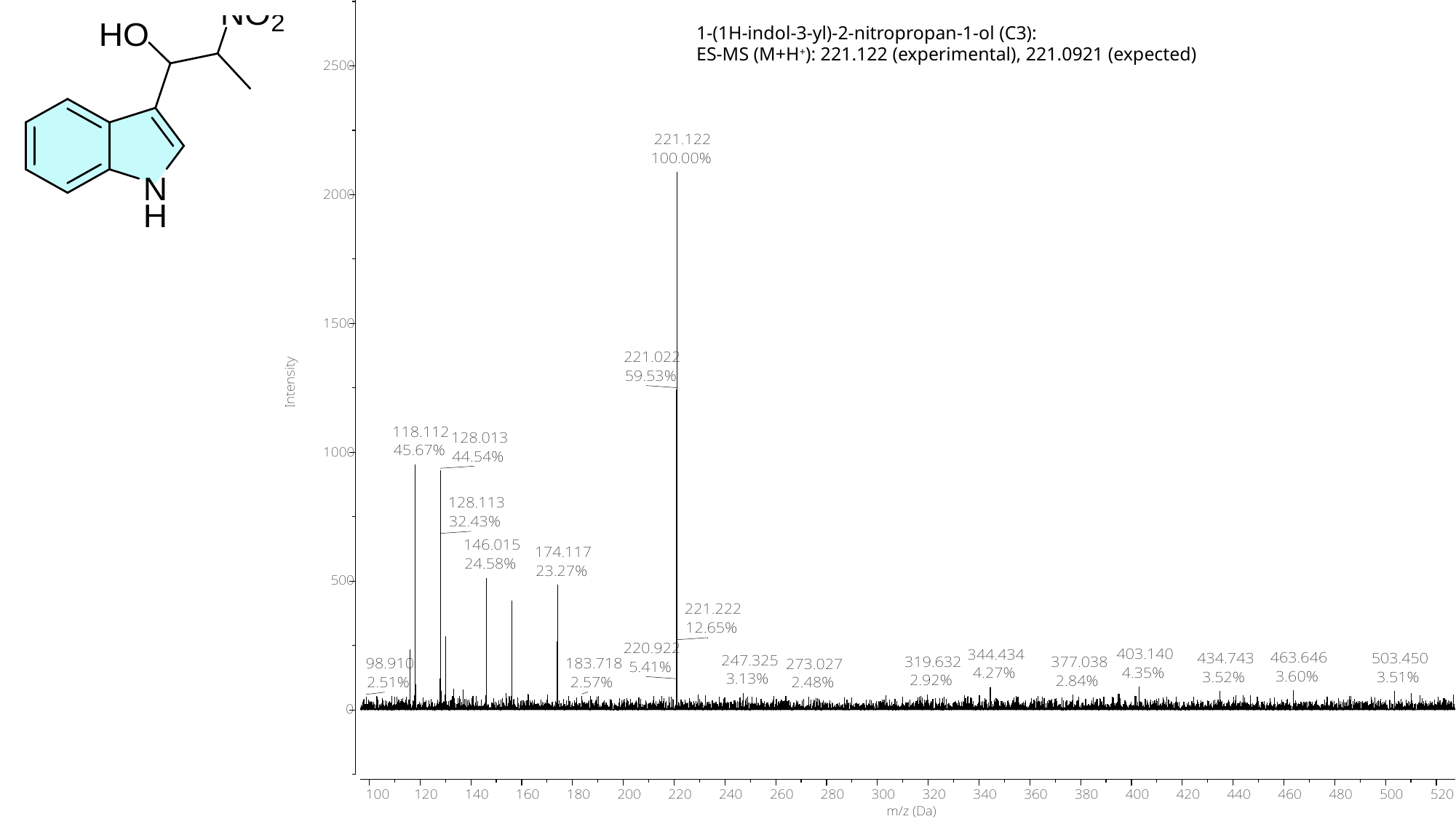

1-(1H-indol-3-yl)-2-nitropropan-1-ol (C3):
ES-MS (M+H+): 221.122 (experimental), 221.0921 (expected)

## Slide 3
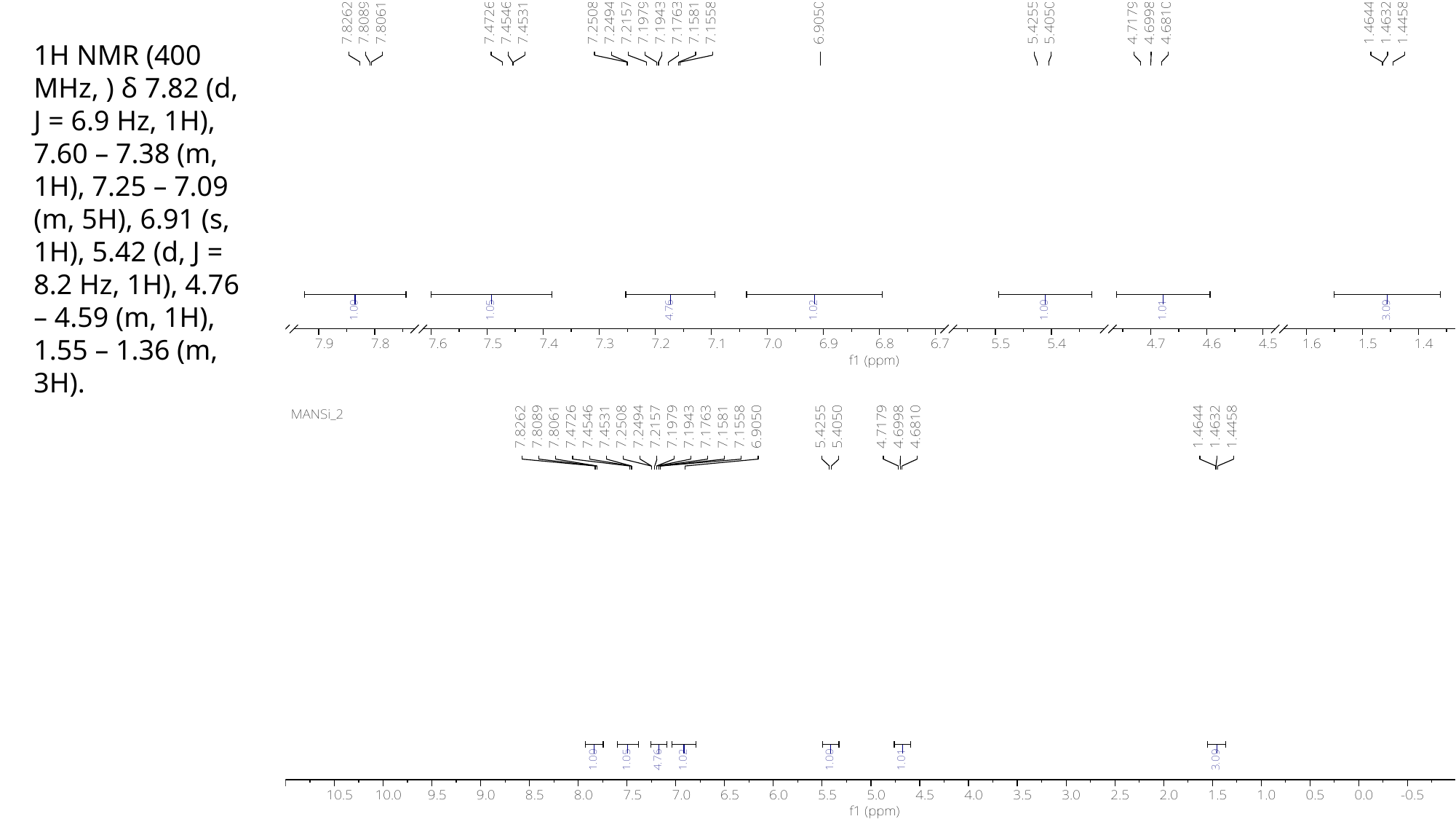

1H NMR (400 MHz, ) δ 7.82 (d, J = 6.9 Hz, 1H), 7.60 – 7.38 (m, 1H), 7.25 – 7.09 (m, 5H), 6.91 (s, 1H), 5.42 (d, J = 8.2 Hz, 1H), 4.76 – 4.59 (m, 1H), 1.55 – 1.36 (m, 3H).

## Slide 4
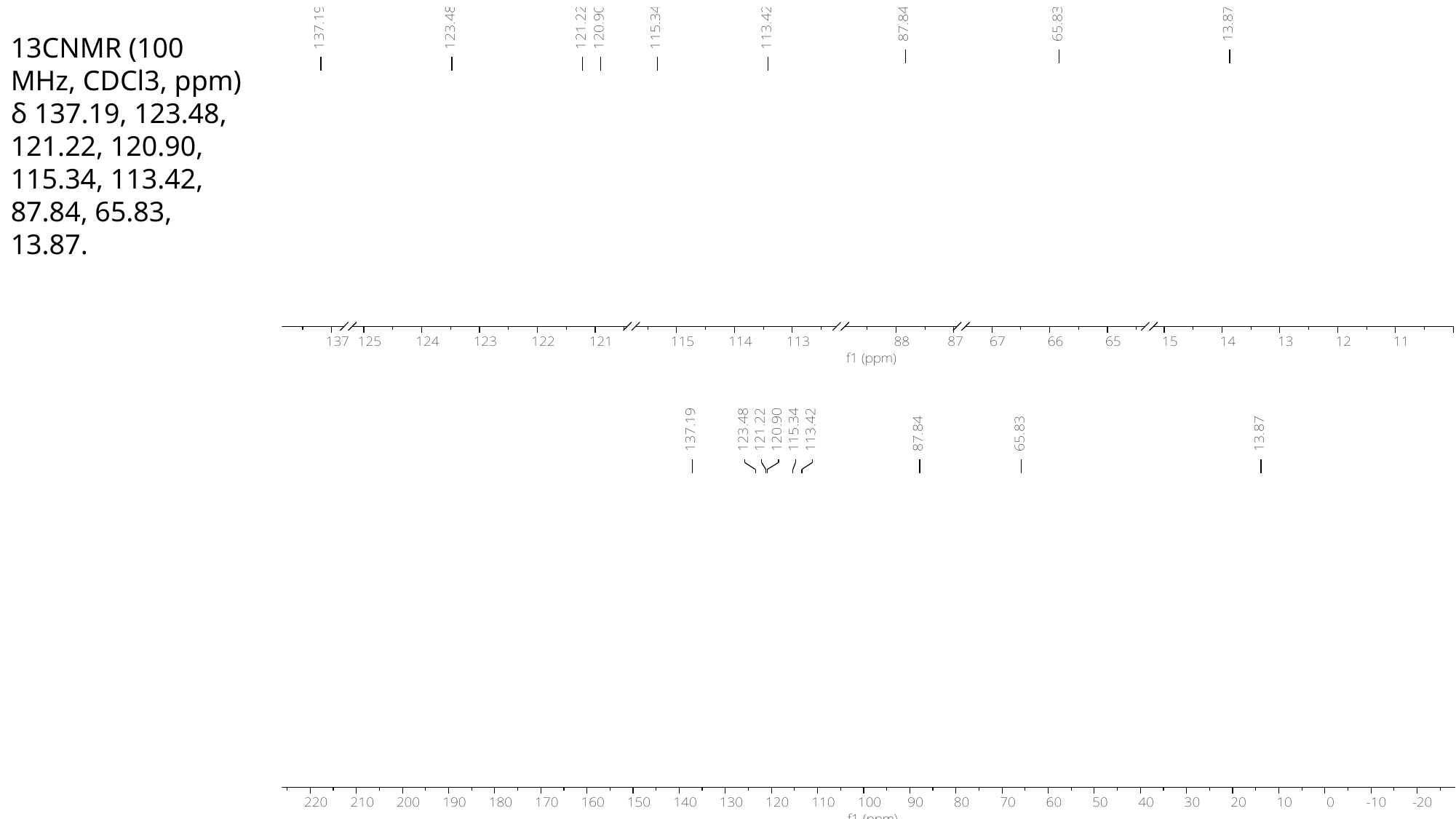

13CNMR (100 MHz, CDCl3, ppm) δ 137.19, 123.48, 121.22, 120.90, 115.34, 113.42, 87.84, 65.83, 13.87.

## Slide 5
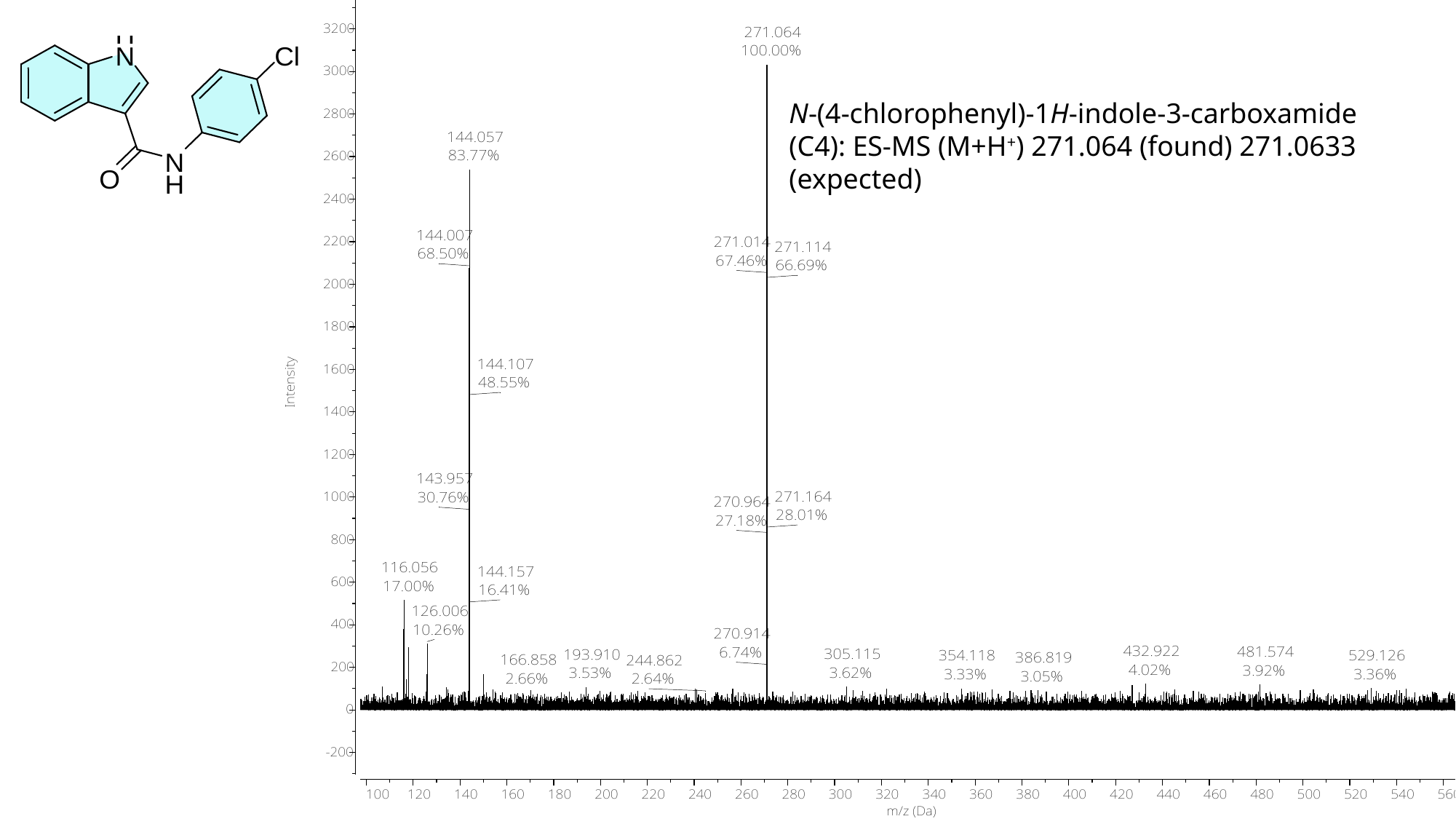

N-(4-chlorophenyl)-1H-indole-3-carboxamide (C4): ES-MS (M+H+) 271.064 (found) 271.0633 (expected)

## Slide 6
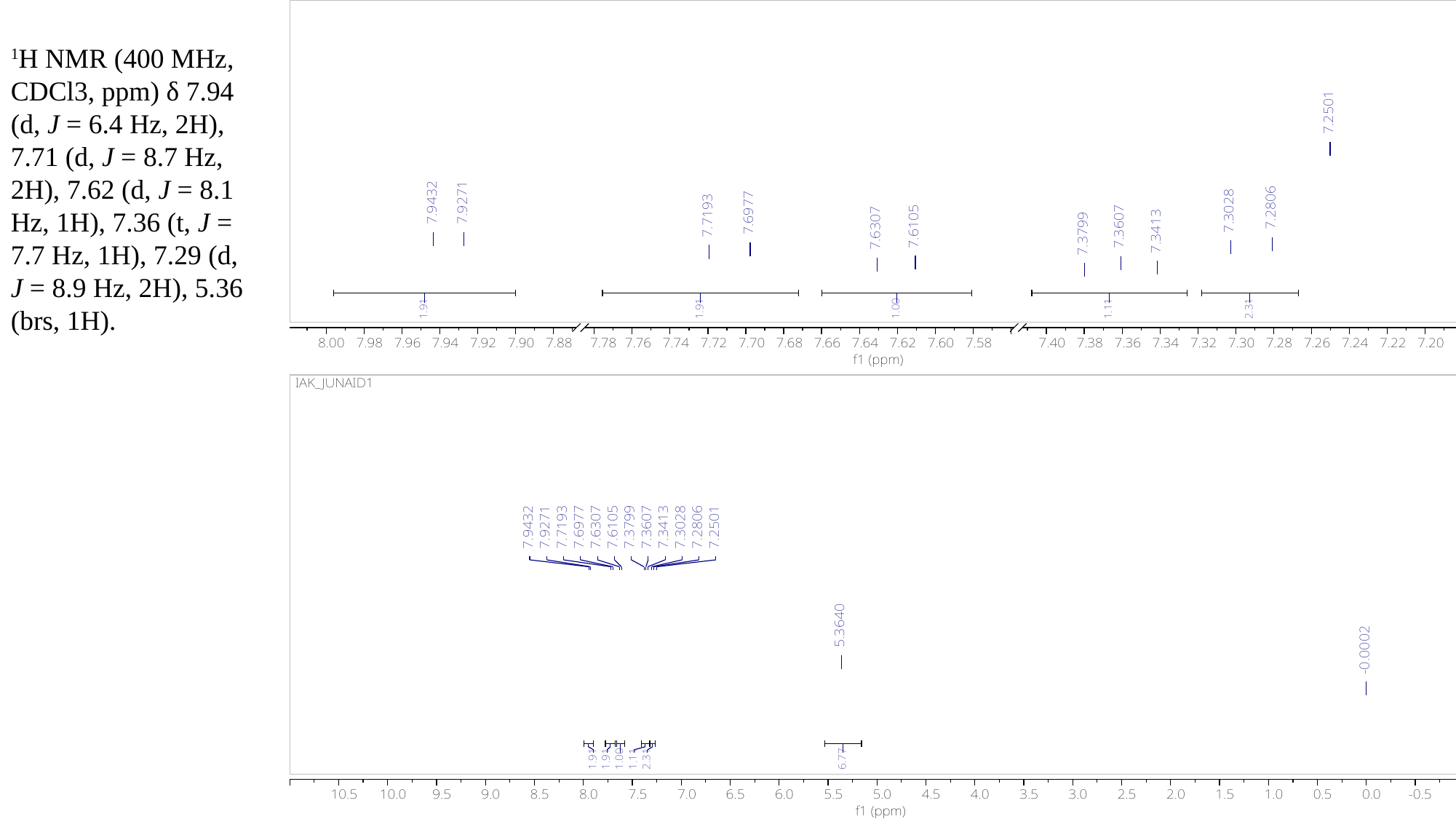

1H NMR (400 MHz, CDCl3, ppm) δ 7.94 (d, J = 6.4 Hz, 2H), 7.71 (d, J = 8.7 Hz, 2H), 7.62 (d, J = 8.1 Hz, 1H), 7.36 (t, J = 7.7 Hz, 1H), 7.29 (d, J = 8.9 Hz, 2H), 5.36 (brs, 1H).

## Slide 7
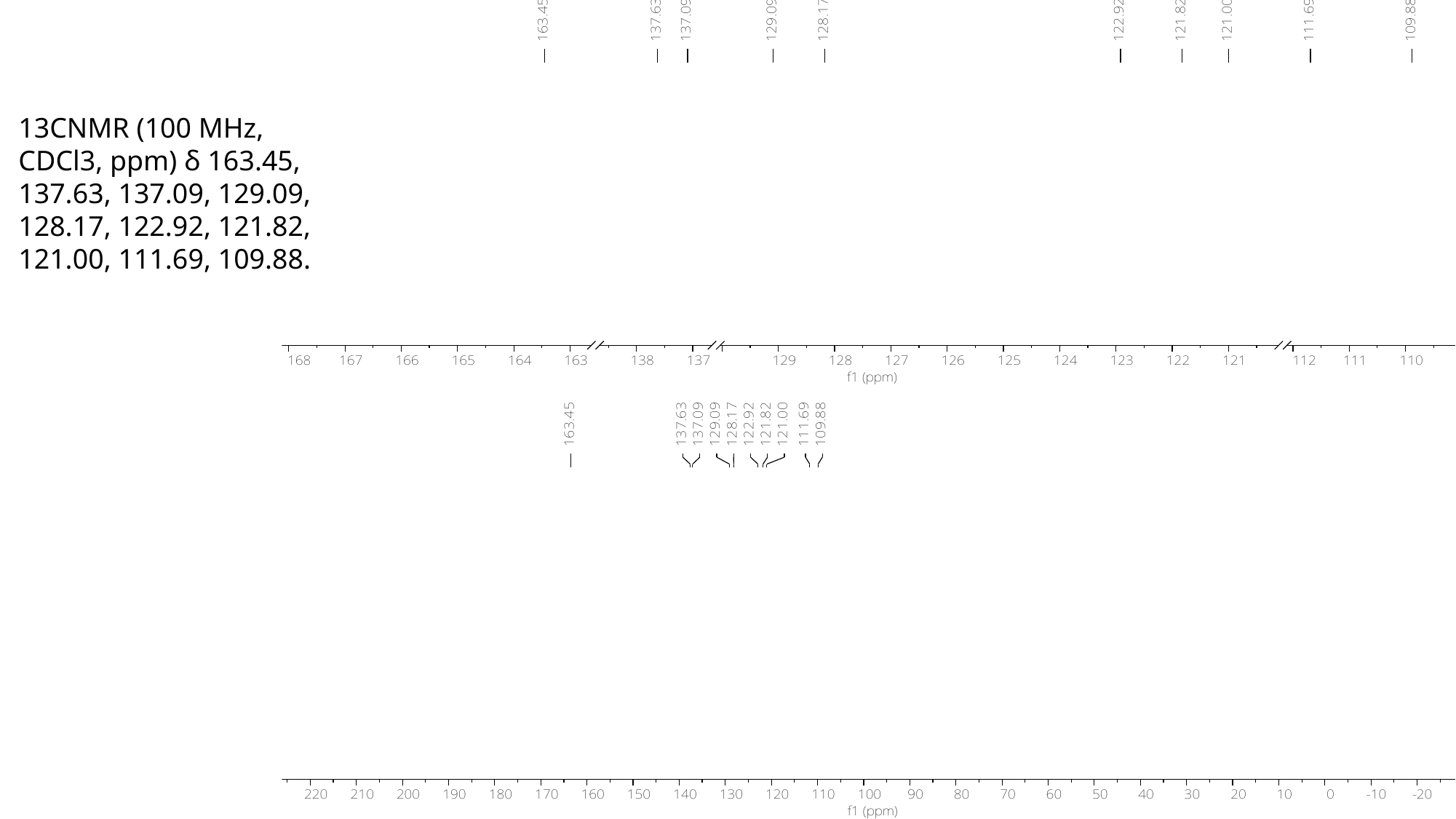

13CNMR (100 MHz, CDCl3, ppm) δ 163.45, 137.63, 137.09, 129.09, 128.17, 122.92, 121.82, 121.00, 111.69, 109.88.

## Slide 8
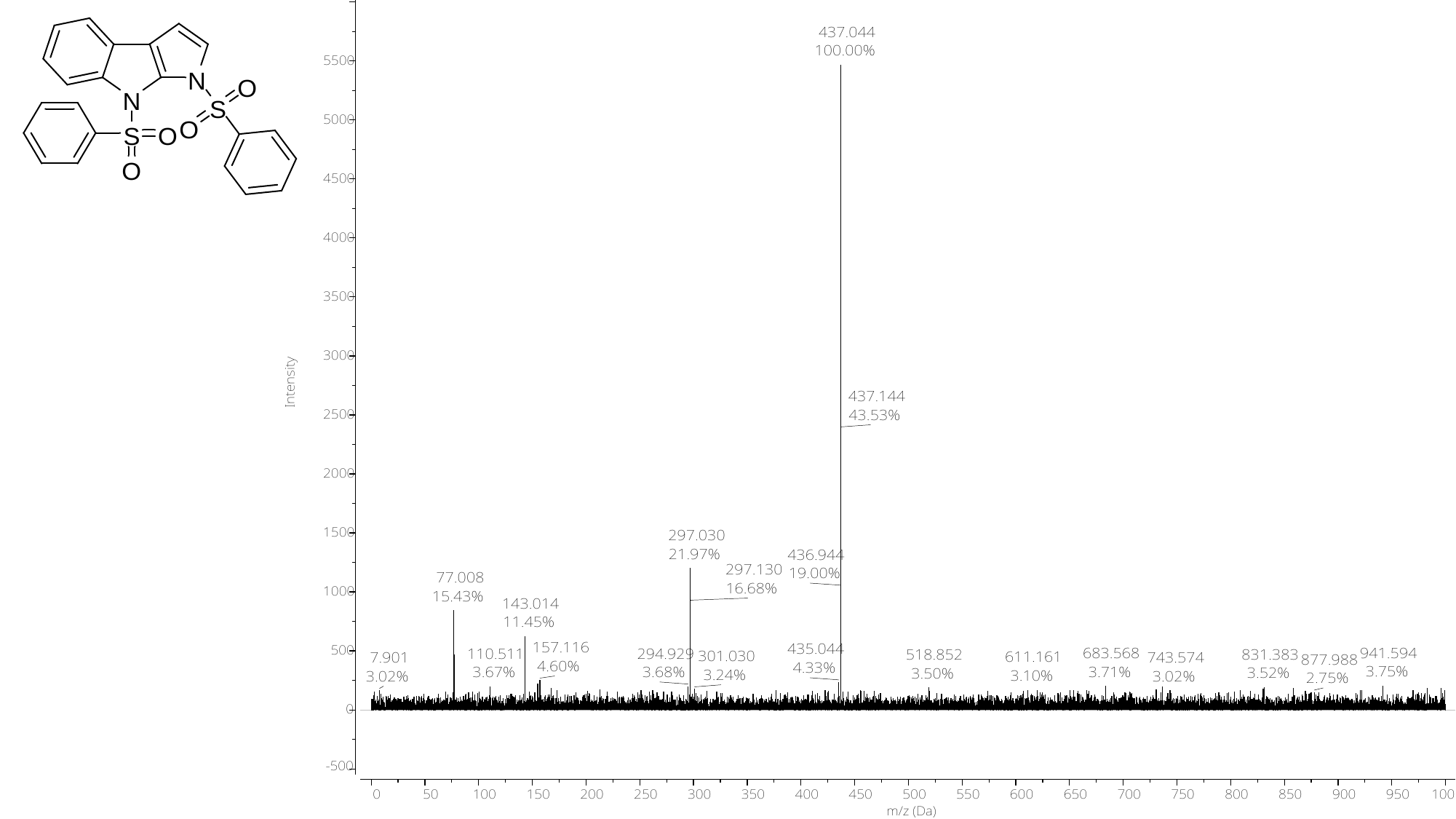

## Slide 9
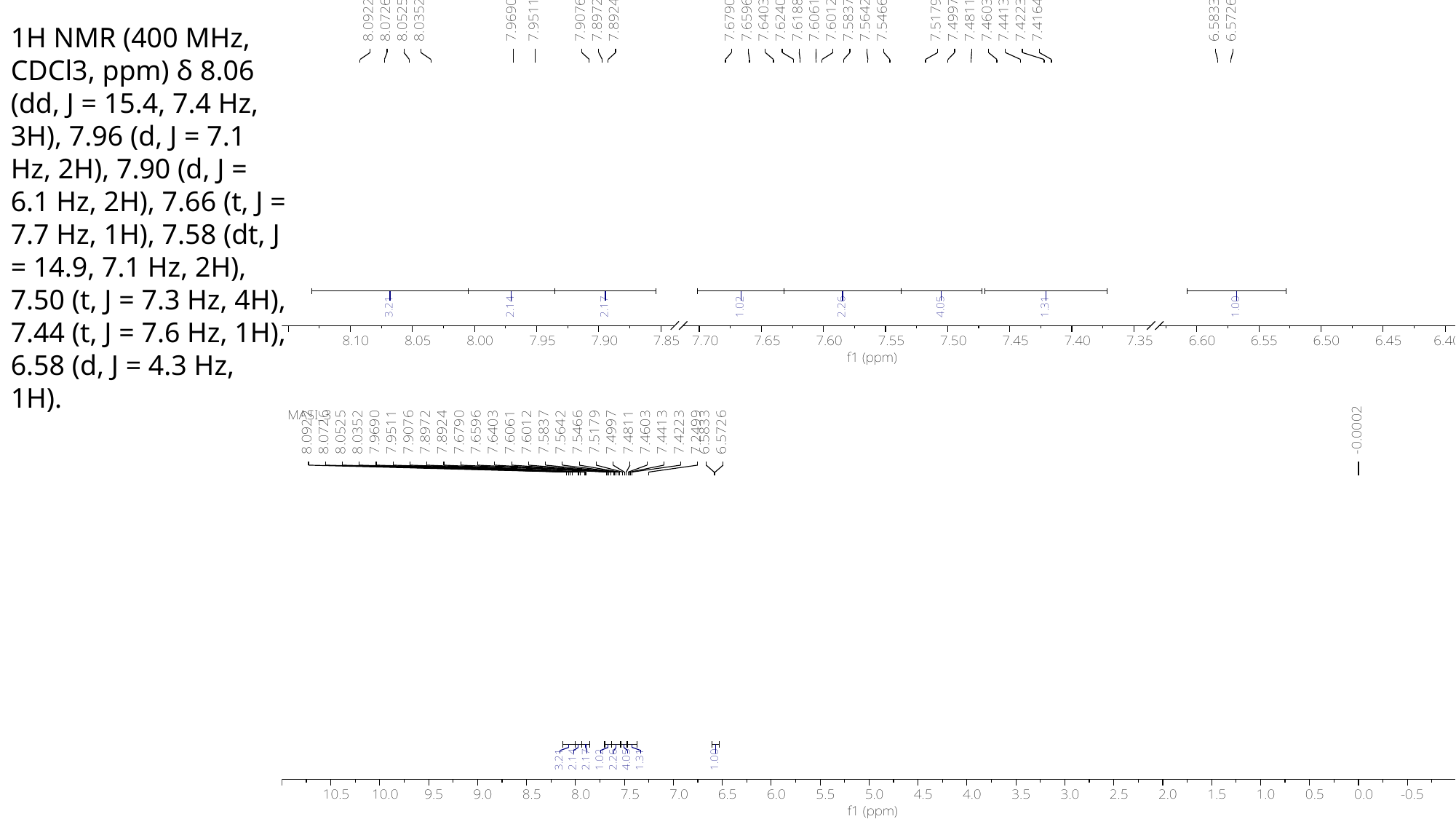

1H NMR (400 MHz, CDCl3, ppm) δ 8.06 (dd, J = 15.4, 7.4 Hz, 3H), 7.96 (d, J = 7.1 Hz, 2H), 7.90 (d, J = 6.1 Hz, 2H), 7.66 (t, J = 7.7 Hz, 1H), 7.58 (dt, J = 14.9, 7.1 Hz, 2H), 7.50 (t, J = 7.3 Hz, 4H), 7.44 (t, J = 7.6 Hz, 1H), 6.58 (d, J = 4.3 Hz, 1H).

## Slide 10
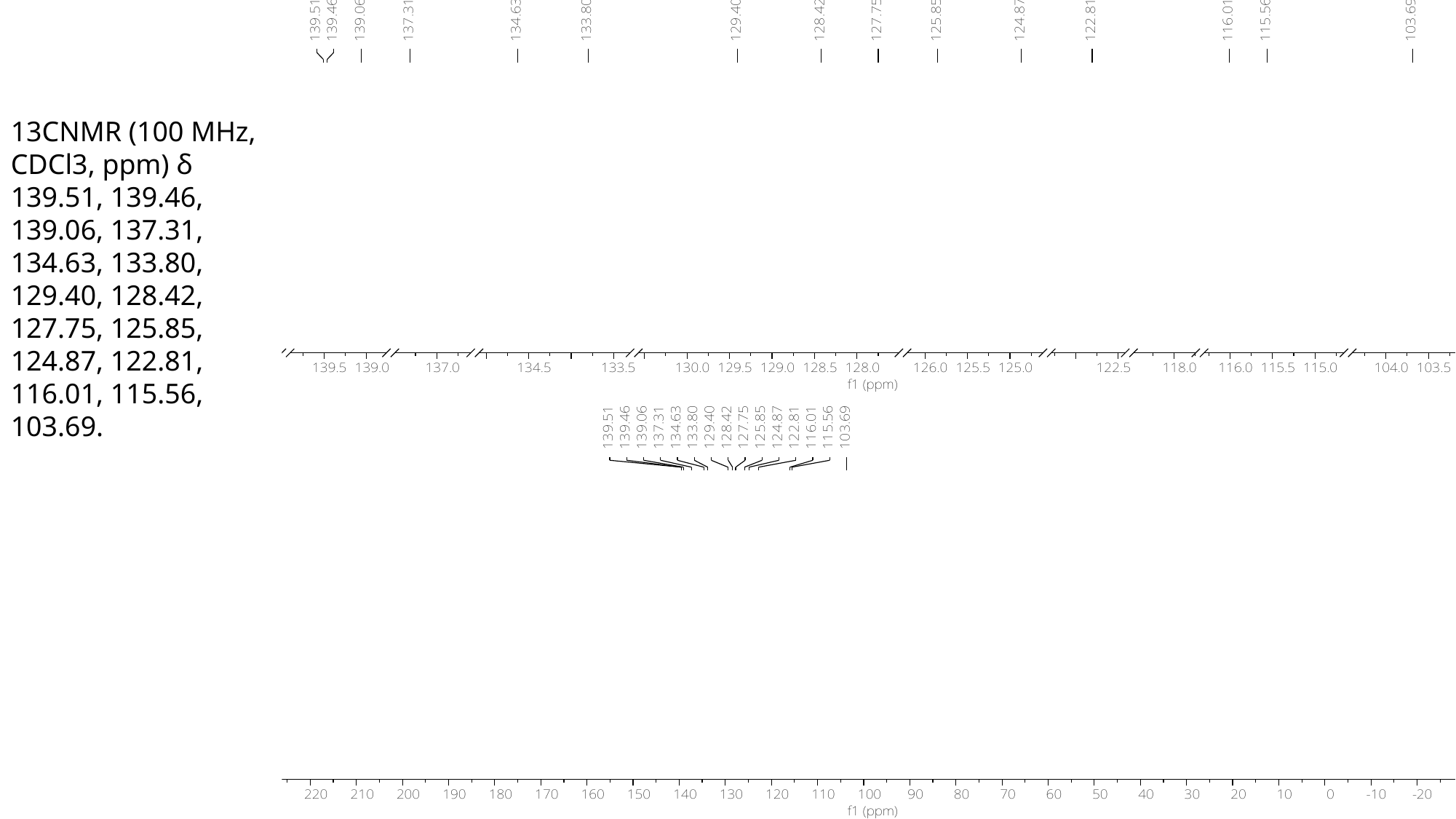

13CNMR (100 MHz, CDCl3, ppm) δ 139.51, 139.46, 139.06, 137.31, 134.63, 133.80, 129.40, 128.42, 127.75, 125.85, 124.87, 122.81, 116.01, 115.56, 103.69.

## Slide 11
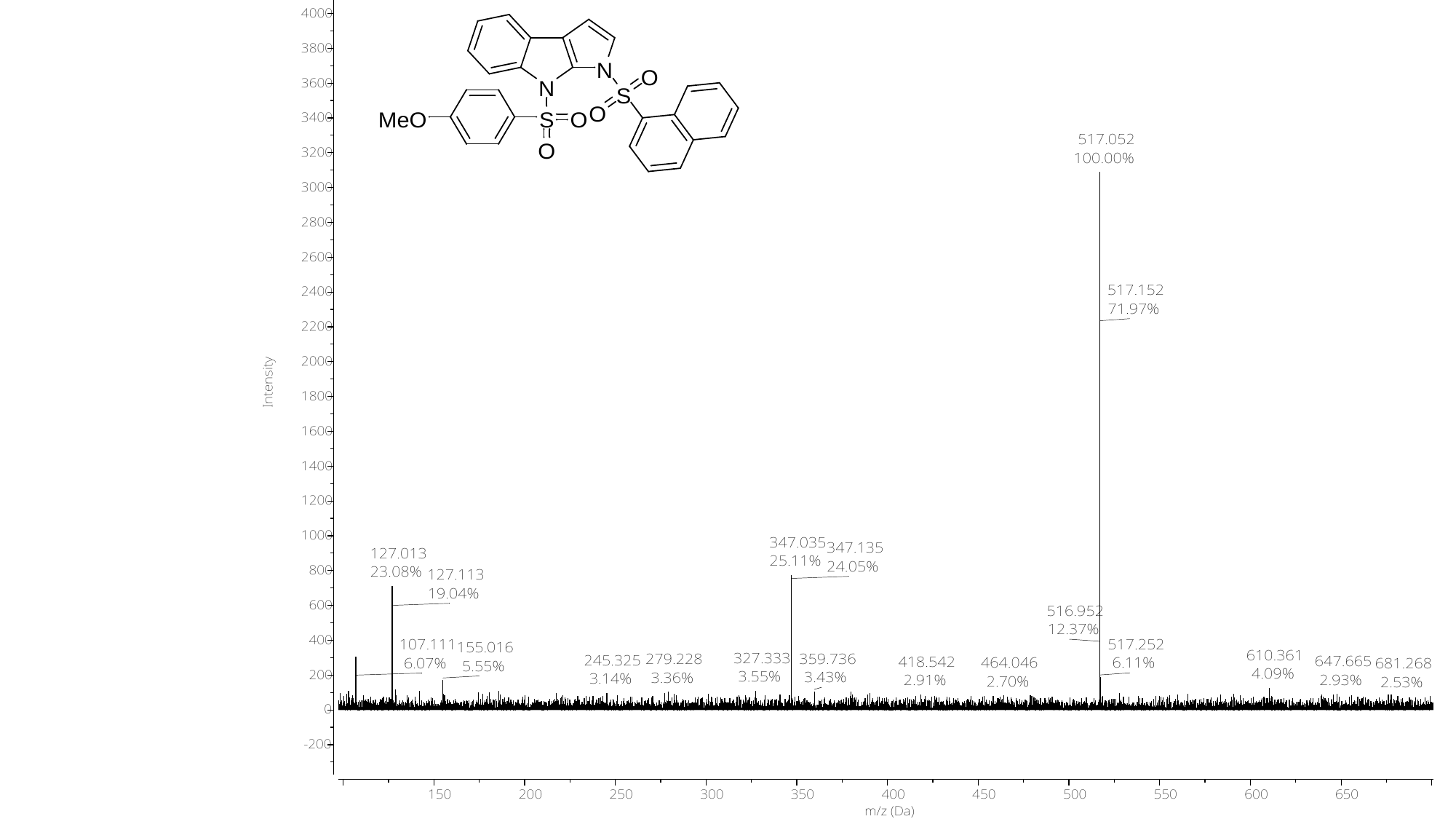

## Slide 12
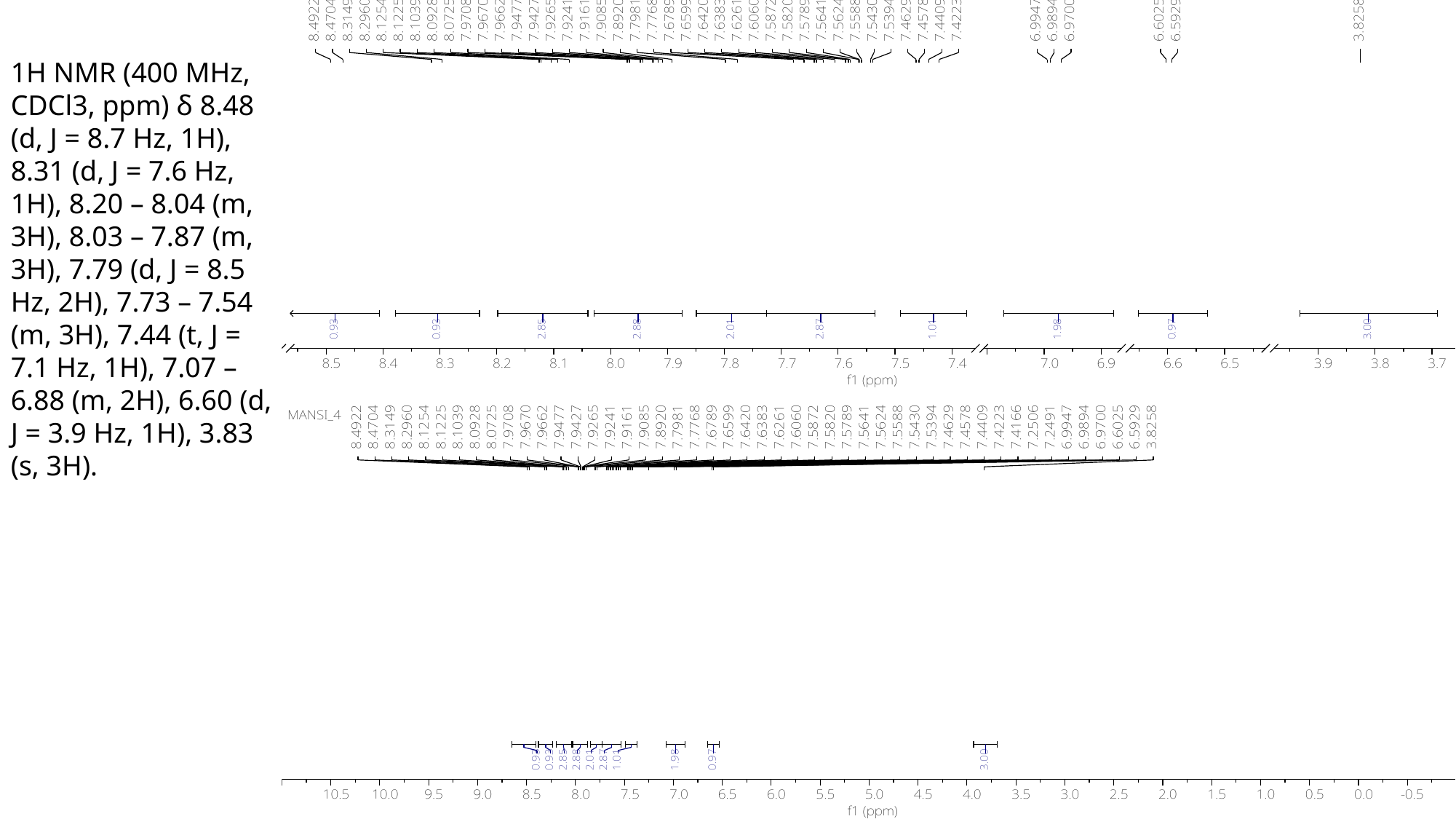

1H NMR (400 MHz, CDCl3, ppm) δ 8.48 (d, J = 8.7 Hz, 1H), 8.31 (d, J = 7.6 Hz, 1H), 8.20 – 8.04 (m, 3H), 8.03 – 7.87 (m, 3H), 7.79 (d, J = 8.5 Hz, 2H), 7.73 – 7.54 (m, 3H), 7.44 (t, J = 7.1 Hz, 1H), 7.07 – 6.88 (m, 2H), 6.60 (d, J = 3.9 Hz, 1H), 3.83 (s, 3H).

## Slide 13
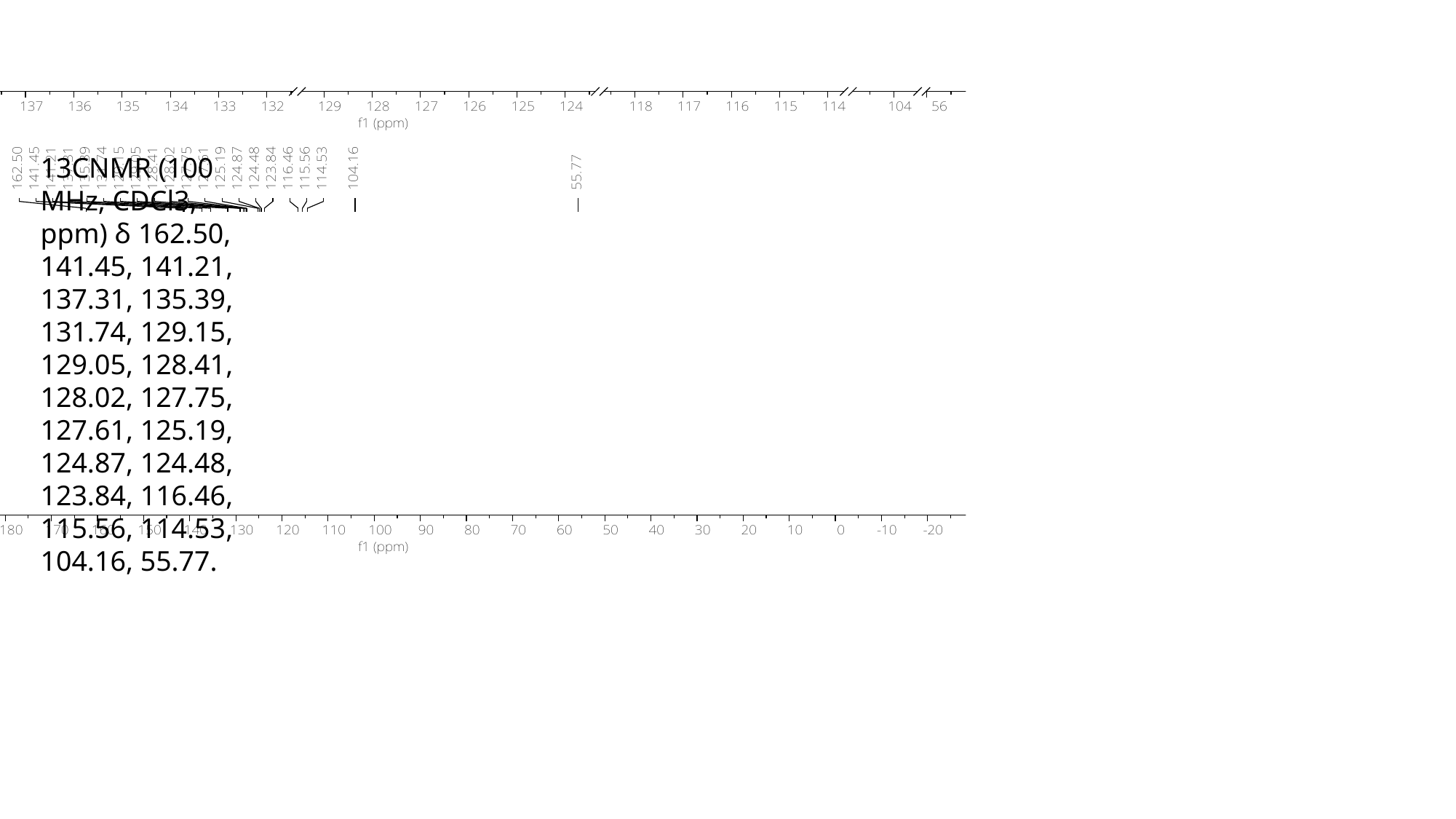

13CNMR (100 MHz, CDCl3, ppm) δ 162.50, 141.45, 141.21, 137.31, 135.39, 131.74, 129.15, 129.05, 128.41, 128.02, 127.75, 127.61, 125.19, 124.87, 124.48, 123.84, 116.46, 115.56, 114.53, 104.16, 55.77.
